# A non-linear pharmacokinetic-pharmacodynamic relationship of metformin in healthy volunteers: An open-label, parallel group, randomized clinical study

**DOI:** 10.1371/journal.pone.0191258

**Published:** 2018-01-17

**Authors:** Hyewon Chung, Jaeseong Oh, Seo Hyun Yoon, Kyung-Sang Yu, Joo-Youn Cho, Jae-Yong Chung

**Affiliations:** 1 Department of Clinical Pharmacology and Therapeutics, Seoul National University College of Medicine, Seoul, Korea; 2 Department of Clinical Pharmacology and Toxicology, Korea University Guro Hospital, Seoul, Korea; 3 Department of Clinical Pharmacology and Therapeutics, Seoul National University Hospital, Seoul, Korea; 4 Clinical Trials Center, Seoul National University Bundang Hospital, Seongnam, Korea; University of Ottawa, CANADA

## Abstract

**Background:**

The aim of this study was to explore the pharmacokinetic-pharmacodynamic (PK-PD) relationship of metformin on glucose levels after the administration of 250 mg and 1000 mg of metformin in healthy volunteers.

**Methods:**

A total of 20 healthy male volunteers were randomized to receive two doses of either a low dose (375 mg followed by 250 mg) or a high dose (1000 mg followed by 1000 mg) of metformin at 12-h intervals. The pharmacodynamics of metformin was assessed using oral glucose tolerance tests before and after metformin administration. The PK parameters after the second dose were evaluated through noncompartmental analyses. Four single nucleotide polymorphisms in MATE1, MATE2-K, and OCT2 were genotyped, and their effects on PK characteristics were additionally evaluated.

**Results:**

The plasma exposure of metformin increased as the metformin dose increased. The mean values for the area under the concentration-time curve from dosing to 12 hours post-dose (AUC_0-12h_) were 3160.4 and 8808.2 h·μg/L for the low- and high-dose groups, respectively. Non-linear relationships were found between the glucose-lowering effect and PK parameters with a significant inverse trend at high metformin exposure. The PK parameters were comparable among subjects with the genetic polymorphisms.

**Conclusions:**

This study showed a non-linear PK-PD relationship on plasma glucose levels after the administration of metformin. The inverse relationship between systemic exposure and the glucose-lowering effect at a high exposure indicates a possible role for the intestines as an action site for metformin.

**Trial registration:**

ClinicalTrials.gov NCT02712619

## Introduction

Metformin is the preferred initial pharmacological agent for type 2 diabetes mellitus [1]. The starting daily dose of metformin is 500 mg, and can be increased to 2550 mg if required based on clinical efficacy and renal function [2]. Previous dose-response studies for metformin have reported a positive relationship, but results are inconsistent when this drug is administered at a high dose. One study with type 2 diabetes patients reported a dose-dependent change in fasting plasma glucose after 24 weeks of treatment with metformin at 1500 mg and 3000 mg per day [3]. In contrast, another study reported stepwise reductions in fasting blood glucose and the area under the curve for glucose when metformin was dosed at 500 or 1500 mg per day, but there was no further significant reduction at 3000 mg per day [4]. Furthermore, in a study that used a dose range of 500 to 2500 mg per day, the maximal response occurred at 2000 mg, not at 2500 mg [5].

The metformin dose-response relationship has been extensively evaluated, yet few reports have explored the pharmacokinetic-pharmacodynamic (PK-PD) relationship of metformin. PK-PD models, such as the indirect response model, which describes the inhibitory effect of metformin on plasma glucose concentrations, have been used in both healthy volunteers and patients with diabetes mellitus [6, 7]. The models in these studies, however, were based on data obtained from studies consisting of a single dose group: 500 mg for healthy volunteers and 850 mg for patients. Because metformin exhibits non-linear PK characteristics and its mechanism of action is not yet fully understood, its PK-PD relationship at either lower or higher doses cannot be extrapolated from the currently available data.

Genetic polymorphisms in several transporters located on renal tubular cells, such as MATE1, MATE2-K, and OCT2, contribute to the large inter-individual variability in pharmacokinetics and pharmacodynamics that has been observed for metformin. The MATE1 (c.922-158G>A, rs2289669) polymorphism was shown to affect the exposure of the drug and to improve its glucose-lowering effect [8, 9]. In addition, a previous study identified common promotor variants of MATE-2K in Korean populations, and a significant increase in renal clearance was observed in subjects with these polymorphisms (c.-396G>A, rs34834489; c.-130G>A, rs12943590) [10]. The effect of the OCT2 (c.808G>T, rs316019) polymorphism on the pharmacokinetics of metformin is controversial, with one article demonstrating its association with high exposure to metformin and another reporting no association [11, 12].

Based on the results from the previous studies mentioned, the aim of this study was to explore the PK-PD relationship of metformin at two doses (250 mg or 1000 mg) on glucose levels. In addition, the effects of genetic polymorphisms on PK characteristics were retrospectively evaluated.

## Methods

### Subjects

This study was approved by the institutional review board of Seoul National University Bundang Hospital (SNUBH) prior to the study on April 7, 2014 (approval number: B-1403/241-003). Healthy male volunteers aged 20 to 45 years old who provided written informed consent underwent a screening procedure that included clinical laboratory tests, electrocardiography, and a physical examination with a survey of their medical history. Based on the results of these procedures, 20 subjects without any significant renal, gastrointestinal, hepatic, or metabolic conditions, including glucose intolerance, were enrolled. Because this study was exploratory in nature, a formal sample size justification was not determined based on a pre-specified power calculation. All study procedures including subject recruitment were done between May 7 and 23, 2014. The study was retrospectively registered at ClinicalTrials.gov (NCT02712619), being not mandatory before recruitment. The authors confirm that all ongoing and related trials for this study are registered.

### Study design

This was a single-centre, randomized, open label, parallel group study that consisted of two doses of metformin (Diabex Tab, Daewoong Pharmaceutical Co.; Seoul, Korea) administered with a 12 hour interval.

After the investigator had confirmed the eligibility, the subjects were allocated to either low-dose group or high-dose group in 1:1 ratio according to the pre-determined sequence generated using SAS 9.3 (SAS Institute Inc. Cary, NC, USA). The subjects were admitted to the Clinical Trial Center of SNUBH the day before the baseline oral glucose tolerance test (OGTT) was performed and remained on a regular diet to ensure a 10 hour overnight fasting state before the OGTT. On day 1, during the baseline OGTT, glucose (75 g) was administered orally, and serial blood samples were collected at 0 (pre-glucose), 0.25, 0.5, 0.75, 1, 1.5, 2, 2.5, and 3 hours after glucose intake. On the evening of day 1, the first dose of metformin (375 mg for the low dose group or 1000 mg for the high dose group) was administered 2 hours after dinner. The next morning (day 2), while in a fasting state, the second dose of metformin (250 mg for the low dose group or 1000 mg for the high dose group) was administered 12 hours after the first dose. Two hours after the second dose of metformin, OGTT was performed as on day 1. For the PK analysis, blood samples were collected at 0 (pre-dose), 0.5, 1, 1.5, 2, 2.5, 3, 4, 6, 8, 10, and 12 hours post-dose, and urine was collected for 12 hours after the second dose.

The first dose (375 mg) in the low-dose group was higher than the second dose (250 mg) in order to simulate a steady state exposure of metformin. The dose in the high-dose group remained at 1000 mg for each dose to ensure safety of volunteers.

### Determination of metformin concentration

The concentrations of metformin in the plasma and urine were determined using liquid chromatography-electrospray ionization-tandem mass spectrometry (LC-ESI-MS/MS, Agilent 1260 HPLC system and Agilent 6490 Triple Quadrupole; Agilent Technologies, Santa Clara, CA, USA). Blood samples collected for PK analyses were centrifuged at 2095 *g* for 10 minutes at 4°C to separate plasma. Plasma (50 μL) and urine (20 μL) samples were mixed with 450 μL and 980 μL of internal standard working solution (phenformin, 50 μg/L in 100% acetonitrile), respectively. After the solution was vortexed for 10 minutes and centrifuged at 18341 *g* for 10 minutes at 4°C, the supernatant (100 μL) was transferred to an autosampling vial. The injection volume was 1 μL. The column used was a Kinetex HILIC column (50 × 2.1 mm, 5 μm; Phenomenex, Torrance, CA, USA) at 24°C. The mobile phase A consisted of 5 mM ammonium formate in distilled water (pH 6.2) and mobile phase B 100% acetonitrile (30:70, v/v). The calibration curves were linear over a range of 10–5,000 μg/L for plasma and 100–25,000 μg/L for urine. The intra- and inter-batch precisions of QC samples were < 9.003% and < 15.57% (at LLOQ level) for plasma and urine, respectively. The mean accuracy values were within ±4.0% and ±14.4% of nominal values for plasma and urine, respectively.

### Pharmacokinetic and pharmacodynamic analysis

PK and PD parameters were calculated using the noncompartmental method in Phoenix^™^ WinNonlin^®^ 6.3 (Certara, St Louis, MO, USA). Among the PK parameters that were evaluated, the maximum plasma concentration (C_max_) and time to reach C_max_ (T_max_) were directly determined using the observed individual time-concentration profiles. The terminal elimination half-life (t_1/2_) was calculated as the natural logarithm of 2 divided by λ_z_, which was defined as the estimated terminal elimination rate constant. The area under the concentration-time curve from dosing to 12 hours post-dose (AUC_0-12_) was calculated using the linear-up/log-down trapezoidal method [13]. Renal clearance (CL_R_) was calculated as the total amount of metformin secreted in the urine during the 12-hour period divided by the AUC_0-12h_.

The evaluated PD parameters included the maximum serum glucose concentration (G_max_), the 2-hour post-OGTT glucose level (PP2), and the area under the glucose concentration-time curve for 3 hours from glucose administration (AUG_0-3h_), which was calculated using the linear trapezoidal rule [14]. To assess the glucose-lowering effect of metformin, the reduction and percent change from the OGTT performed before metformin administration were calculated.

### Genotyping

Four single nucleotide polymorphisms, including MATE1 (c.922-158G>A, rs2289669), MATE2K (c.-396G>A, rs34834489; and c.-130G>A, rs12943590), and OCT2 (c.808G>T, rs316019) were analysed. TaqMan allelic discrimination assays were performed using an ABI Prism 7500 Sequence Detection System (Thermo Fisher Scientific) and the results were used for genotyping.

### Statistical analysis

All statistical analyses were performed using SAS 9.3 (SAS Institute Inc. Cary, NC, USA). Demographic, PK, and PD variables were summarized as descriptive statistics, and Mann-Whitney U tests were used to compare values between the two dose groups. PK-PD relationships were assessed using linear regression using least-squares method and E_max_ model [15]. In all cases, p-values lower than 0.05 were considered to indicate statistical significance.

## Results

**Fig 1:**
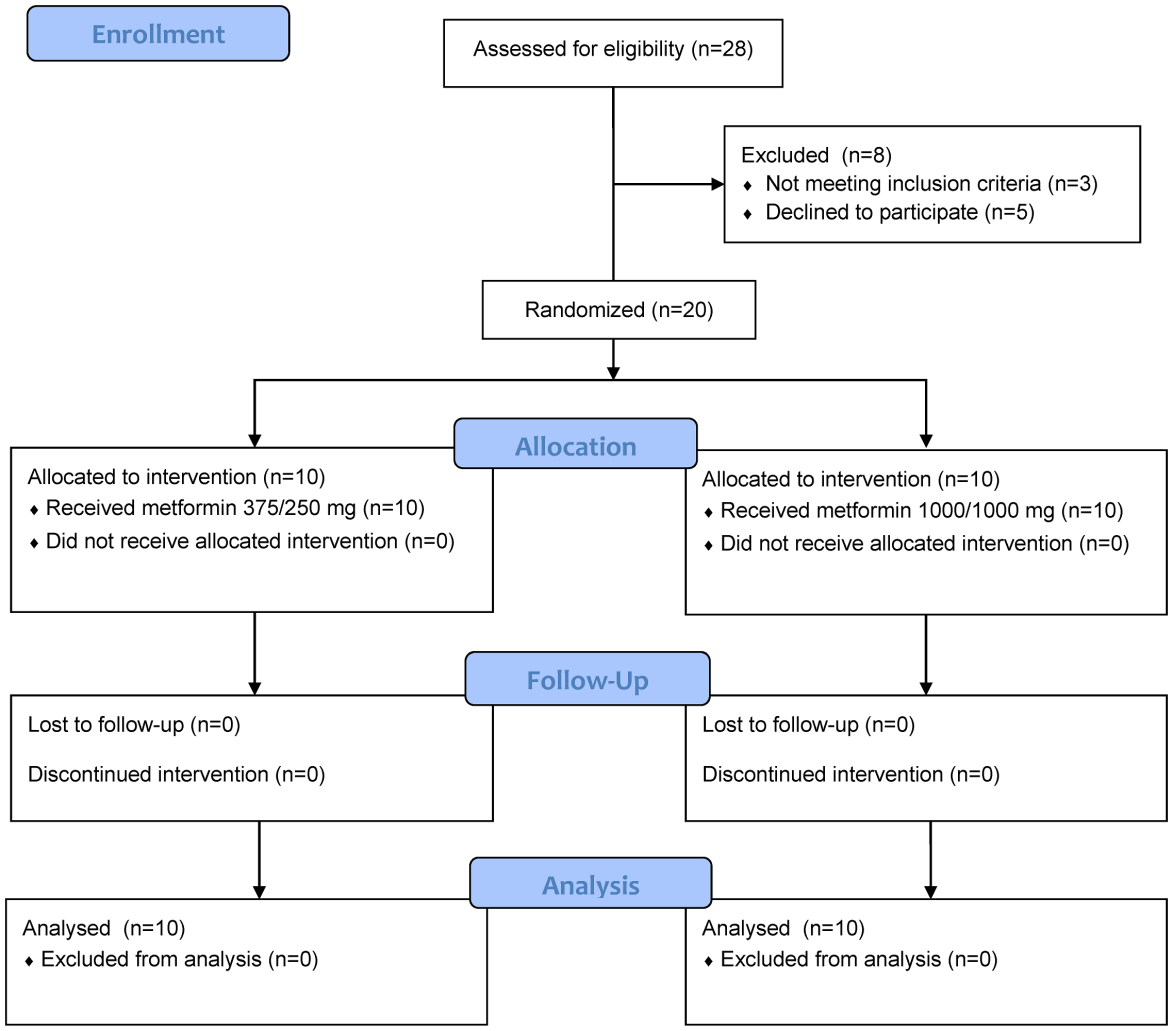
CONSORT flowchart.

A total of 20 healthy male subjects were randomly assigned to either the low dose (n = 10) or the high dose (n = 10) group, and all of them completed the study (Fig 1). The mean ages (24.5 versus 25.0 years old) and weights (66.8 versus 69.7 kg) of the subjects were similar between the low dose group and the high dose group. No subjects had a history or showed evidence of renal disease, and all displayed normal creatinine clearance when calculated with Cockcroft-Gault formula with a mean value of 126 and 124 mL/min in the low dose group and high dose group, respectively (*p* = 0.69).

**Fig 2:**
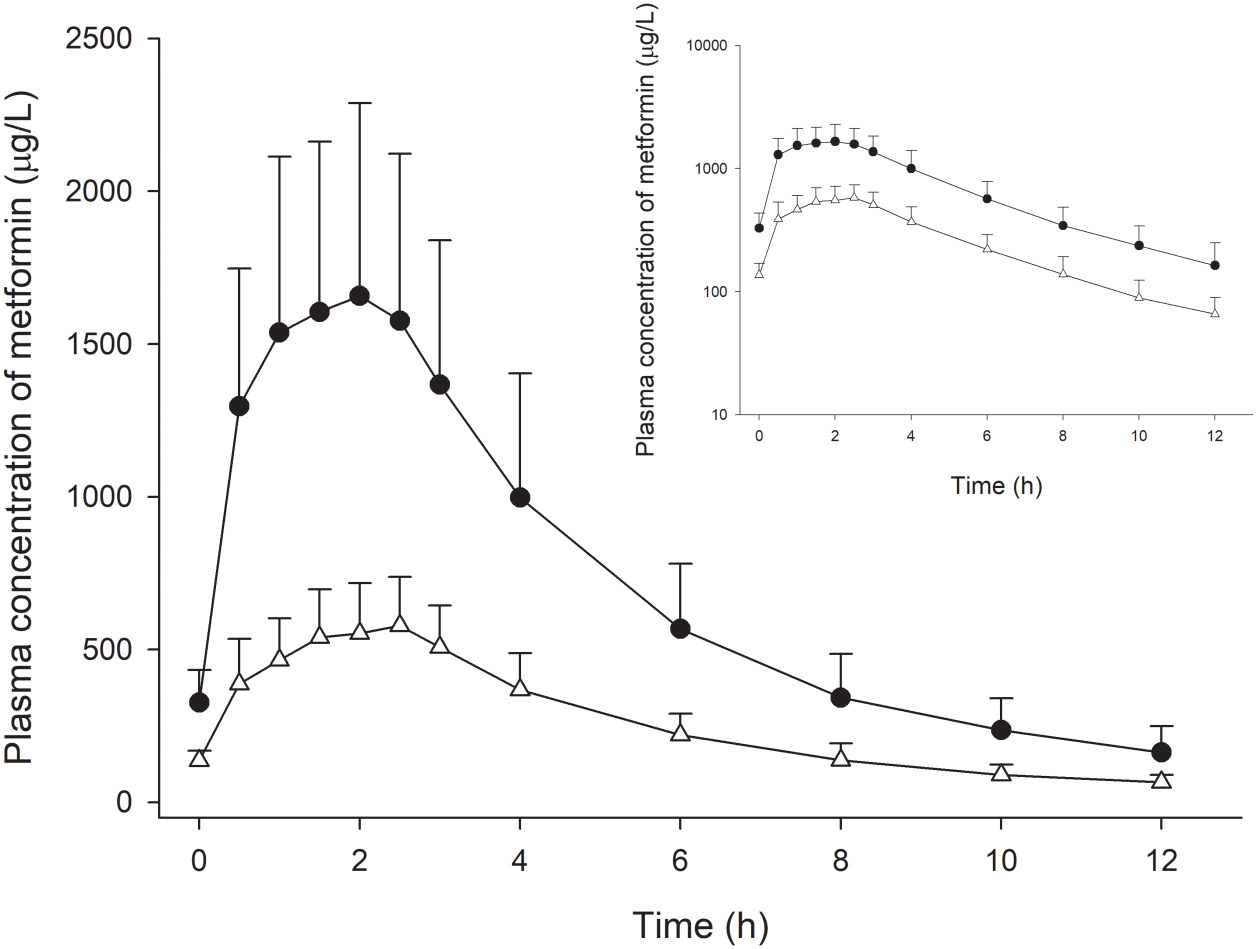
Mean concentration profile of metformin administered at 250 mg (Δ) and 1000 mg (●). (Inlet: log-linear scale; error bars present standard deviation).

The PK parameters and time-concentration profile of metformin after administration are shown in Table 1 and Fig 2, respectively. The Mann-Whitney U tests showed no statistically significant difference for T_max_ and t_1/2_ between the two dose groups. Furthermore, no pre-dose PD variables during the OGTTs were significantly different between the two dose groups. While the median absolute change from baseline was larger for all evaluated parameters except for PP2 in the high-dose group, none of the values were statistically different between the two groups (Table 2, S1 Fig).

**Table 1:** Pharmacokinetic parameters after metformin administration.

	250 mg (N = 10)	1000 mg (N = 10)	p-value^a^
C_max_ (μg/L)	591.7 (247.5)	1937.5 (863.0)	0.0002
AUC_0-12h_ (h·μg/L)	3168.7 (1140.4)	9621.0 (5237.6)	0.0002
CL_R_ (mL/min)	571.2 (56.5)	430.3 (113.1)	0.0004
t_1/2_ (h)	3.3 (1.1)	3.1 (1.5)	0.7055
T_max_ (h)	2.0 (1.0)	1.5 (1.0)	0.1118

^a^: Mann-Whitney U test

The data are presented as medians (interquartile ranges). C_max_, maximum plasma concentration; AUC_0-12_, area under the concentration-time curve from dosing to 12 hour postdose; CL_R_, renal clearance; t_1/2_, terminal elimination half-life; T_max_, time to reach C_max_

**Table 2:** Results of oral glucose tolerance test (OGTT) before and after administration of metformin.

	250 mg		1000 mg	
	Predose	Postdose	Predose	Postdose
AUG_0-3h_ (h·mg/dL)	391.2 (19.8)	366.0 (36.3)	397.8 (67.8)	370.3 (39.6)
Reduction in AUG_0-3h_ (h·mg/dL)	30.6 (25.0)		41.9 (60.1)	
% change in AUG_0-3h_ (%)	-7.1 (7.0)		-10.8 (12.2)	
G_max_ (mg/dL)	169.5 (48.0)	157.0 (22.0)	174.5 (39.0)	145.5 (22.0)
Reduction in G_max_ (mg/dL)	11.5 (31.0)		29.0 (30.0)	
% change in G_max_ (%)	-7.6 (17.5)		-18.1 (11.2)	
PP2 (mg/dL)	132.5 (19.0)	111.5 (19.0)	139.0 (38.0)	125.5 (31.0)
Reduction in PP2 (mg/dL)	23.5 (10.0)		16.5 (22.0)	
% change in PP2 (%)	-16.5 (8.7)		-12.8 (15.5)	

The data are presented as the medians (interquartile ranges). AUG_0-3h_, area under the glucose concentration-time curve for 3 hours from glucose administration; G_max_, maximum serum glucose concentration; PP2, 2-hour-post-OGTT glucose level

**Fig 3:**
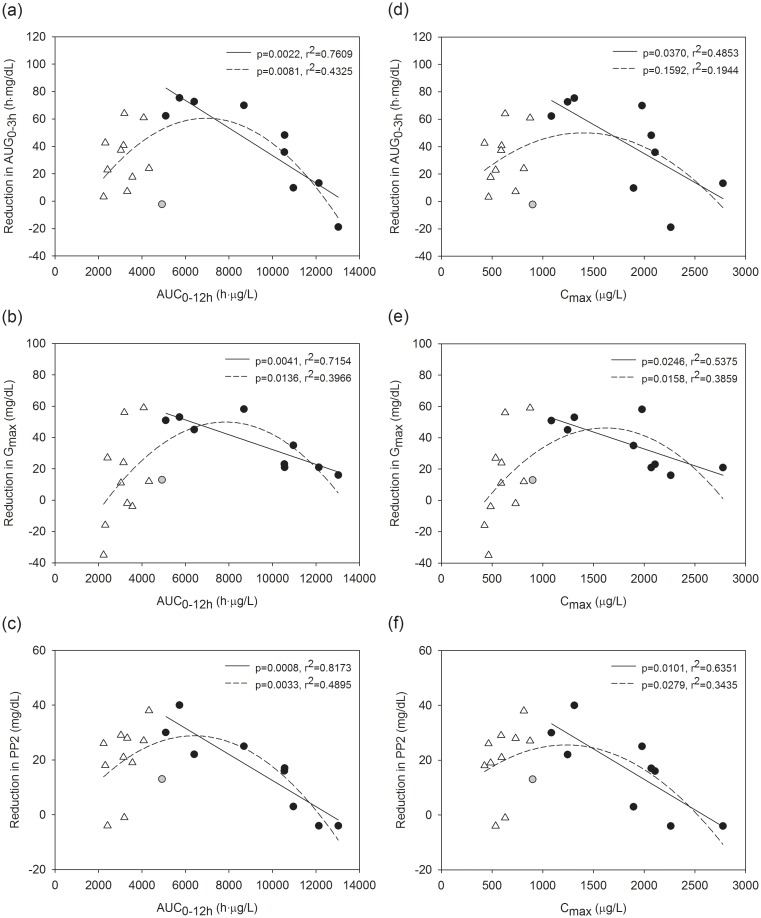
Relationships between pharmacokinetic-pharmacodynamic parameters after metformin administration at 250 mg (triangle) and 1000 mg (circle). Reduction in (a) AUG_0-3h_, (b) G_max_, and (c) PP2 versus AUC_0-12h_; reduction in (d) AUG_0-3h_, (e) G_max_, and (f) PP2 versus C_max_. (The solid lines and dashed lines represent the linear and quadratic models, respectively. The black circle represents data included in the linear regression at high exposure. P-values are from the F-tests, and r^2^ represents the coefficient of determination.).

Non-linear exposure-response relationships on glucose levels were observed for metformin in this study. The polynomial regression with quadratic models were significant to the entire dataset, except between C_max_ and AUG_0-3h_, including high-dose and low-dose groups together. In addition, there were significant inverse linear relationships between the reduction in OGTT parameters and the PK parameters in the high-dose group when the C_max_ and AUC_0-12h_ exceeded 901 to 1086 μg/L and 4919 to 5100 h·μg/L, respectively. AUC_0-12h_ accounted for 72% to 82% of the variation in PD parameters, while C_max_ accounted for 49% to 64% (Fig 3). Non-linear exposure-response relationships with inverse relationships at high exposure were also observed when percent changes in PD parameters were used to explore the relationship (S2 Fig). The data could not be adequately explained with the E_max_ model, and when the linear regression included all subjects in the high-dose group, the models were not significant.

Although the number of subjects in each genotype were too small to provide adequate power for testing for significant differences, the PK parameters were comparable among subjects with the genetic polymorphisms (MATE1, MATE2K, and OCT2) analysed in this study (S1 Table).

## Discussion

In this study, the PK and PD characteristics of metformin at 250 or 1000 mg were evaluated. A non-linear relationship was observed between metformin systemic exposure and its glucose-lowering effect. Significantly, this study provides the first set of data supporting an inverse relationship between systemic exposure to metformin and its glucose-lowering effects at high exposure. The findings of this study are in partial agreement with those of a previous dose-response study which reported a higher mean change from baseline in fasting plasma glucose and HbA1c at a daily dose of 2000 mg metformin as compared to a 2500 mg dose [5]. This study, however, was limited by the different dosing scheme between the two groups: the low-dose group received an initial 375-mg dose followed by a dose of 250 mg, whereas the high-dose group received two doses of 1000 mg. The initial 375-mg dose followed by a dose of 250 mg in the low-dose group was designed to achieve an exposure similar to that expected at steady state after 250 mg administration, considering the chronic use of metformin. Compared to a previous multiple-dose study of metformin 250 mg twice daily, the mean C_max_ was similar (640.5 versus 614.7 μg/L) and the AUC_0-12h_ was lower (4153 versus 3160 h·μg/L) in the low-dose group of our study [16]. For the high-dose group, the C_max_ and AUC_0-12h_ were comparable to those achieved during the dosing interval at steady state with 500 to 850 mg twice daily [17, 18]. Although the increased first dose in the low-dose group may have led to enhanced PK or PD effects which is in contrast to the equal first dose in the high-dose group, the PK-PD relationship trend could be explored because the doses and exposure in the low-dose group were apparently lower than those in the high-dose group.

A possible hypothesis that explains the inverse relationship between systemic exposure to metformin and its glucose-lowering effect stems from possible unabsorbed metformin in the intestinal tract. Because the fraction of metformin that is metabolized during a first pass is negligible, it is likely that subjects in the high dose group with lower systemic exposure to metformin had a higher amount of metformin remaining unabsorbed in the intestinal tract. Consequently, the unabsorbed portion of metformin may exert a local anti-glycaemic effect on the intestines in addition to its systemic effect, which might have reached a plateau after exposure to a high dose. Although the mechanism for local anti-glycaemic effect in intestines remains unclear, the time point of the OGTT performed in our study (a 3-h measurement beginning 2 h after metformin administration) supports the hypothesis by reflecting the unabsorbed metformin in the small intestine and colon [19]. In diabetic rats, the intraduodenal administration of metformin produced a larger response than intraportal or intravenous administration, suggesting that presystemic biophases contribute to its overall glucose-lowering effect [20]. Another study in rats reported that intraduodenal infusions of metformin activated duodenal AMPK and lowered hepatic glucose production and plasma glucose levels, while a portal vein infusion of metformin did not [21]. Furthermore, a recent study in humans reported that delayed-release metformin was more effective than similar doses of a more bioavailable extended-released form of metformin, indicating the role of the gut in the glucose-lowering effect of metformin [22].

Because more than one-third of all patients fail to achieve glycaemic goals while being administered metformin monotherapy, PK or PD variability should be considered when attempting to understand the clinical effect of the drug [23]. Genetic polymorphisms are one set of factors that can affect the PK or PD characteristics of metformin. Because we did not stratify pharmacogenetics characteristics between groups, we were unable to demonstrate whether there was any significant association between the pharmacokinetics of metformin and the single nucleotide polymorphisms MATE1, MATE2-K, and OCT2. In addition to the renal transporters that were assessed in this study, in hepatocyte, OCT1 is known for its effect on responses to metformin in healthy volunteers and in patients with diabetes [24–26]. Genetic variants of transcription factors or proteins that are involved in the pharmacological activity of metformin, such as calpain 10, may also affect the pharmacodynamics of metformin [27, 28]. It is possible that these genetic polymorphisms, whether they were or were not evaluated in this study, affected the results of our analysis of the PK-PD relationships in combination.

The healthy subjects who were enrolled in this study were different from patients who have diabetes mellitus because healthy subjects can be assumed to have normal glucose levels. One concern that might arise from this difference is that a maximal drug effect might be masked due to homeostatic gluconeogenesis in healthy subjects. In our study, it is possible that the 3 subjects with the highest metformin exposure had low reductions in OGTT parameters because they had low glucose levels at baseline. The mean baseline AUG_0-3h_ for these 3 subjects was 353.3 h·mg/dL while that for the remainder of the subjects in the high-dose group was 426.1 h·mg/dL. Thus, we additionally explored the PK-PD relationship using % changes from baseline in AUG_0-3h_, G_max_, and PP2 to incorporate the differences in baseline values. The relationships were similar to the results shown in Fig 3, supporting non-linearity with an inverse trend when metformin exposure was high (S2 Fig). Future studies with balanced predose PD characteristics according to the exposure or a PK-PD modelling approach would be useful for the precise determination of the PK-PD relationship.

## Conclusions

In conclusion, we report the existence of non-linearity in the PK-PD relationship of metformin (250 and 1000 mg) to glucose levels in healthy subjects. Further investigations into the mechanism of action of metformin and pharmacogenetic characteristics that might affect its pharmacodynamics will increase our understanding of PK-PD relationships.

## Supporting information

S1 Table Renal clearance (mL/min) according to the OCT2, MATE1, and MATE2K genotypes. (DOCX)

S1 Fig Mean glucose profile during oral glucose tolerance tests before (▲, ●) and after (Δ, ○) administration of metformin (a: 250 mg, b: 1000 mg; error bars present the standard deviation). (TIF)

S2 Fig Relationships between pharmacokinetic-pharmacodynamic parameters after metformin administration at 250 mg (triangle) and 1000 mg (circle) (% change in (a) AUG_0-3h_, (b) G_max_, and (c) PP2 versus AUC_0-12h_; % change in (d) AUG_0-3h_, (e) G_max_, and (f) PP2 versus C_max_. The solid lines and dashed lines represent the linear and quadratic models, respectively. The black circle represents data included in the linear regression at high exposure).
(TIF)

S1 File CONSORT checklist. (PDF)

S2 File Study protocol. (PDF)

S3 File Pharmacokinetic and pharmacodynamic dataset. (ZIP)
