# Supplementary material for: A non-linear pharmacokinetic-pharmacodynamic relationship of metformin in healthy volunteers: An open-label, parallel group, randomized clinical study
Source: PLoS One. 2018 Jan 17;13(1):e0191258. doi: 10.1371/journal.pone.0191258 (PMC5771593; doi:10.1371/journal.pone.0191258)
Supplement: S1 Table — (DOCX) [file pone.0191258.s001.docx]

**S1 Table. Renal clearance (mL/min) according to the OCT2, MATE1, and MATE2K genotypes**

|  | Metformin 250 mg | | | Metformin 1000 mg | | |
| --- | --- | --- | --- | --- | --- | --- |
|  | M/M | M/m | m/m | M/M | M/m | m/m |
| OCT2  (rs316019) | 565.7 (252.4) [9] | 576.8 [1] |  | 419.6 (312.1) [9] | 520.6 [1] |  |
| MATE1  (rs2289669) | 538.4 (93.9) [2] | 565.7 (58.1) [5] | 576.8 (216.7) [3] | 464.3 (111.3) [3] | 419.6 (134.2) [3] | 394.8 (290.9) [4] |
| MATE2K  (rs34834489) | 557.1 (59.9) [2] | 579.2 (214.9) [6] | 527.1 (71.3) [2] | 419.6 (111.3) [3] | 440.9 (312.1) [7] |  |
| MATE2K  (rs12943590) | 545.8 (85.3) [4] | 585.4 (178.1) [5] | 527.1 [1] | 448.6 (177.6) [4] | 440.9 (306.3) [5] | 419.6 [1] |
| Data presented as medians (ranges) [number of subjects].  M: major allele, m: minor allele | | | | | | |
