# Supplementary figures and images for: A non-linear pharmacokinetic-pharmacodynamic relationship of metformin in healthy volunteers: An open-label, parallel group, randomized clinical study
Source: PLoS One. 2018 Jan 17;13(1):e0191258. doi: 10.1371/journal.pone.0191258 (PMC5771593; doi:10.1371/journal.pone.0191258)

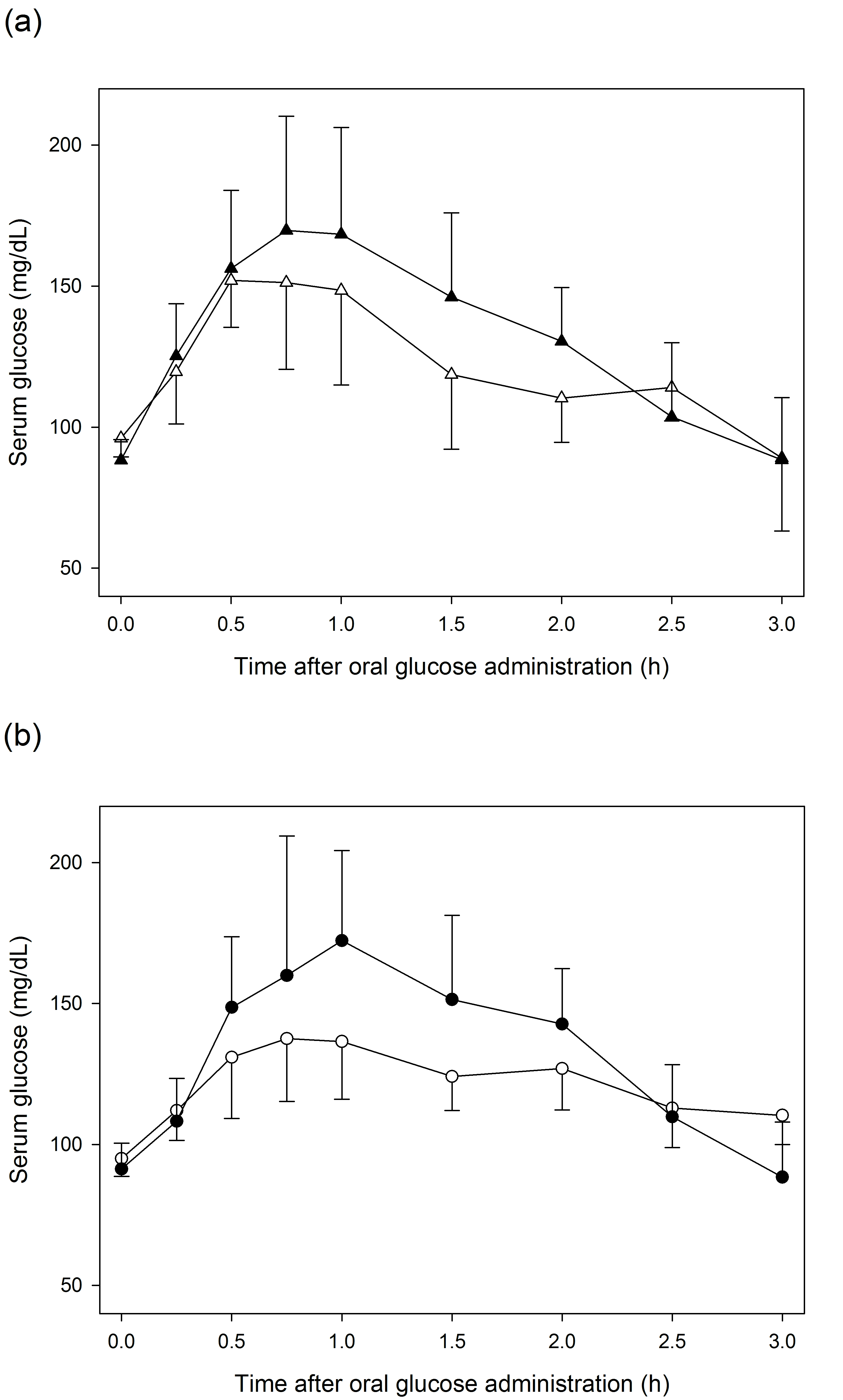

Supplement: S1 Fig — (TIF) [file pone.0191258.s002.TIF]

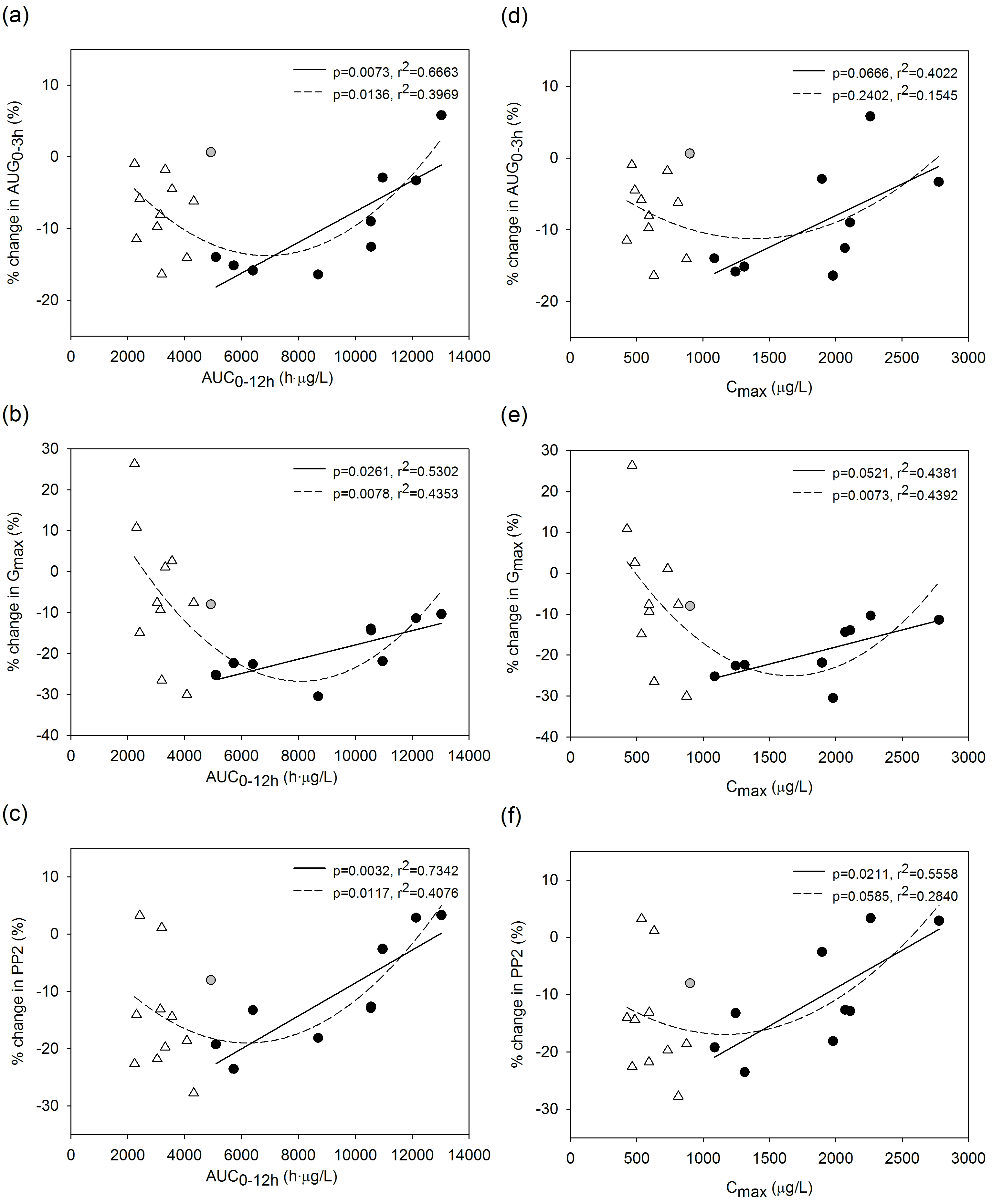

Supplement: S2 Fig — The solid lines and dashed lines represent the linear and quadratic models, respectively. The black circle represents data included in the linear regression at high exposure). (TIF) [file pone.0191258.s003.TIF]
