# Supplementary material for: A non-linear pharmacokinetic-pharmacodynamic relationship of metformin in healthy volunteers: An open-label, parallel group, randomized clinical study
Source: PLoS One. 2018 Jan 17;13(1):e0191258. doi: 10.1371/journal.pone.0191258 (PMC5771593; doi:10.1371/journal.pone.0191258)
Supplement: S2 File — (PDF) [file pone.0191258.s005.pdf]

임 상 연 구 계 획 서

(Clinical study protocol)

건강한 남성에서 Metformin 250/1000 mg 투여 시
약동-약력학 특성을 평가하기 위한 연구

An open-label, parallel group clinical trial to investigate the pharmacokinetics/pharmacodynamics of 250/1000 mg of metformin IR after oral administration in healthy male volunteers

Version No. 1.0

분당서울대학교병원 임상시험센터
(Seoul National University Bundang Hospital, Clinical Trial Center)

➢ 임상연구개요

**연구제목 (Study title)**: 건강한 남성에서 Metformin 250/1000 mg 투여 시 약동-약력학 특성을 평가하기 위한 연구 (An open-label, parallel group clinical trial to investigate the pharmacokinetics/pharmacodynamics of 250/1000 mg of metformin IR after oral administration in healthy male volunteers)
**연구책임자 및 기관 (Principal investigator)**: 정재용, 분당서울대학교병원 (Prof. Jae-Yong Chung, Seoul National University Bundang Hospital)
**연구기간 (Study period)**: IRB 승인일~2015.3 (IRB approval ~ 2015.3)<br>**임상연구형태 (Type of study)**: 기타 (Others)
**목적 (Purpose)**<br>건강 성인 남성을 대상으로 서로 다른 두 용량의 메트포민 투여 시의 약동학 및 약력학(혈당강하효과)적 특성을 비교 평가한다.<br>(To investigate the pharmacokinetics/pharmacodynamics of metformin at two different doses in healthy male volunteers)
**연구설계 (Study design)**<br>공개, 단일순서, 약동/약력학 연구<br>(Open-label, single sequence, pharmacokinetic/pharmacodynamic study)
**목표연구대상자수 (Number of subjects)**: 총 20명 (250 mg 군 10명, 1000 mg 군 10명)<br>(A total of 20 subjects (10 subjects for 250 mg arm, 10 subjects for 1000 mg arm))
**연구대상자 등록 (Subject enrollment)**<br>**포함기준 (Inclusion criteria)**<br>1. 한국인 성인으로 만 20~45세, 체중 50~100 kg 이며, Ideal Body Weight ((Height – 100) X 0.9)의 25 % 이내인 남성 (Korean adult in age of 20 to 45 whose weight varies within 25% of their ideal body weight ((Height – 100) X 0.9))<br>2. 스크리닝 검사시 시행한 공복 혈당 검사(FPG)에서 70-125 mg/dL 의 혈당 범위를 보이는 자 (Subject with fasting plasma glucose of 70-125 mg/dL at screening)<br>3. 신뢰할 수 있고, 임상연구 기간 동안 시간을 할애하고 임상연구 계획서를 준수할 의지가 있는 연구대상자 (Subject who are willing to participate and comply with the study protocol)<br>4. 임상연구에 대한 자세한 설명을 듣고 완전히 이해한 후, 자의로 참여를 결정하고 주의사항을 준수하기로 서면 동의한 자 (Subject who understood the study and agreed to the written informed consent)<br>**제외기준 (Exclusion criteria)**<br>1. 임상적으로 유의한 호흡기, 순환기, 신장, 위장관, 간, 내분비, 혈액, 신경 (운동실조 포함), 정신 질환 혹은 기타 만성 질환, 알코올 혹은 약물 중독에 대한 임상적인 증거가 있는 자 (Subject with clinically significant pulmonary, cardiovascular, renal, gastrointestinal, endocrine, hematologic, neurologic, mental, or chronic disease or subjects with evidence of drug abuse)<br>2. 연구약의 흡수에 영향을 줄 수 있는 위장관계 질환(크론씨병, 궤양, 급성 또는 만성 췌

장염 등) 이나 위장관계 수술(단, 단순 맹장수술이나 탈장수술은 제외) 의 과거력이 있는 자 (Subject who has current or history of gastrointestinal disease or surgery which can influence study drug absorption)

3. 임상연구용 의약품의 투약 14일 이내에 유의한 약물상호작용이 알려진 약물과 임상 연구자가 판단하기에 적합하지 않다고 판단되는 약물을 복용한 자. (Subject who took medication which may have significant drug interaction with study drug within 14 days of study drug administration)
4. 임상연구 개시 전 30일 이내 혈액을 공여하거나 60일 이내에 다른 임상연구용의약품 혹은 시판 중인 약물의 임상연구에 참여한 자 (Subject who donated whole blood within 30 days or previously participated in other clinical study within 60 days)
5. 임상연구용 의약품 및 동일계열의 성분을 포함한 약물 및 기타 다른 약물(아스피린, 항생제 등) 에 대한 과민반응 또는 임상적으로 유의한 과민반응의 병력이 있는 자 (Subject with known for hypersensitivity reactions to the study drug)
6. 연구기간 중 임신을 계획하고 있거나 계획 중이 아니어도 인정받는 피임법 (예: 본인 및 파트너의 불임수술, 파트너의 자궁 내 피임기구, 간벽 피임법, 격막 또는 콘돔의 병용)을 사용할 수 없는 자 (Subject who cannot use reliable methods for contraception during the study period)
7. 지속적으로 음주(21 units/week 초과, 1 unit = 10 g of pure alcohol)를 하거나 임상연구기간 중 금주할 수 없는 자 (Subject who drink alcohol more than 21 units/week or who cannot abstain from drinking during the study period)
8. 임상실험실검사 결과를 비롯한 기타 사유로 인하여 연구자가 임상연구 참여에 부적합하다고 판단한 자 (Subject who are considered inadequate based on clinical laboratory tests or investigator’s discretion)

임상연구용 약품명 및 용량 용법 (Study drug and dose):

1. Metformin(250 mg 군): 다이아벡스 정 (대웅) 250 mg 정
   375 mg 1일 1회, 250 mg 1일 1회 씩 총 2회
   (Diabex tablet (Daewoong) 250 mg)
   (A total of 2 doses: 375 mg once daily and 250 mg once daily)
2. Metformin(1000 mg 군): 다이아벡스 정 (대웅) 1000 mg 정
   1000 mg 1일 1회, 1000 mg 1일 1회 씩 총 2회
   (Diabex tablet (Daewoong) 1000 mg)
   (A total of 2 doses: 1000 mg once daily and 1000 mg once daily)

소요기간 (Study period): 3 일 (3 days)

연구방법 (Study methods)

본 연구는 용량군 별로 10명씩 총 20 명의 연구대상자를 대상으로 투약한다.

자원자에 한하여 제 1기 임상연구용의약품 첫 번째 입원 예정일(-1d)로부터 30일 이내(-30d ~ -

1d)에 스크리닝 검사를 시행하여, 본 임상연구에 적합하다고 판단되는 연구대상자를 선정한다.

선정된 연구대상자는 첫 번째 입원 예정일 (-1d) 오후 5시까지 분당서울대병원 임상시험센터에 입원한다. 입원 후 저녁 6시경 임상시험용식사를 한다. 입원 둘째 날 (1d) 오전 기상 후 첫 소변을 수집하며 8시경 유전형 및 대사체 채혈, 안전성 검사를 시행한다. 오전 10시경부터 경구당부하 후 약력학 채혈을 시작하며 마지막 약력학 채혈(오후 1시경) 후 점심식사를 제공한다. 오후 6시경 저녁식사를 제공하며, 오후 8시경 각 용량군 별로 metformin 375 mg 또는 1000 mg을 240 mL의 물과 함께 복용한다. 셋째 날 (2d) 오전 기상 후 첫 소변을 수집한다. 오전 8시경 대사체 채혈 및 투약 전 약동학 채혈을 수행 후 각 용량군 별로 metformin 250 mg 또는 1000 mg을 공복 상태에서 240 mL의 물과 함께 복용한다. 투약 직전 방광을 비운 후 12시간 동안 집뇨를 수행한다. 이후 예정대로 약동학 채혈을 수행하며, 2시간 뒤 오전 10시경부터 경구당부하 후 약력학 채혈을 수행한다. 예정된 약동학 채혈을 모두 수행 후 연구대상자는 오후 8시경 퇴원할 수 있다.

(Ten subjects will be assigned to each treatment group and a total of 20 subjects will be administered. Volunteers will undergo screening test within 30 days from the scheduled admission to assess their eligibility. Eligible subjects will be admitted to the Clinical Trial Center of Seoul National University Bundang Hospital at -1d and have regular dinner. At 1d, Urine and blood sample for genotype, metabolite and safety assessment will be collected from 8 a.m.. Oral glucose tolerance test (OGTT) will be performed at 10 a.m. followed by blood samples to assess pharmacodynamics. Lunch and dinner will be supplied at 1 p.m. and 6 p.m., respectively. Metformin 375 mg or 1000 mg will be administered with 240 mL of water at 8 p.m according to the allocated treatment group. At 2d 8 a.m. on fasting state, metformin 250 mg or 1000 mg will be administered with 240 mL or water after the scheduled pre-dose urine and blood sample collection. Urine and blood sample for pharmacokinetic analysis will be collected until 12 hours post-dose, and OGTT will be performed at 10 a.m. followed by blood samples for pharmacodynamics analysis. The subjects will be discharged after the scheduled sample collection.)

평가기준

- [약동학 지표]: AUC, C_{max}, $t_{1/2}$, CL_R, Fe 및 약동학 구획 모델의 속도상수
- [약력학 지표]: 혈당농도곡선하면적 (AUGC), 최대혈당치(G_{max})
- [탐색적 지표]: 대사체, 유전형별 약동/약력학 차이
- 이러한 지표에 대해 metformin 저용량 투여시와 고용량 투여시 비교분석을 실시 한다.

연구일정요약 (Summary of study procedure)

Period	Day	Planned time	예상 시간 (predicted time)	Event	OGTT[1]	PK[1]	유전형 [2], 대사체검사 (genotype[2], metabolite)	투약 [3] (Drug administration[3])	집뇨 [4] (Urine collection[4])	Serum Crea-tinine	안전성검사 [5] Safety assessment[5]
Screening	-30D ~ -1D			스크리닝 (Screening)							X
Treatment (250 mg/ 1000 mg 용량군)	-1D		17:00	입원 [6] (Admission[6])							
			18:00	저녁식사 (Dinner)							
	1D	0h	8:00	대사체검사 (Metabolite)			X				X
		2h	10:00	OGTT	X						
		2h 15m	10:15		X						
		2h 30m	10:30		X						
		2h 45m	10:45		X						
		3h	11:00		X						
		3h 30m	11:30		X						
		4h	12:00		X						
		4h 30m	12:30		X						
		5h	13:00	점심식사 (OGTT 후) (Lunch after OGTT)	X						
		10h	18:00	저녁식사 (Dinner)							
		12h	20:00	투약 (Administration)				X			

Period	Day	Planned time	예상 시간 (predicted time)	Event	OGTT[1)]	PK[1)]	유전형 2), 대사체검사 (genotype[2)], metabolite)	투약 3) (Drug administration[3)])	집뇨 4) (Urine collection[4)])	Serum Crea-tinine	안전성검사 5) Safety assessment[5)]
	2D	0h	8:00	대사체검사 (Metabolite)		X	X		0~6h	X	
				투약 (Administration)		X		X			
		0h 30m	8:30			X					
		1h	9:00			X					
		1h 30m	9:30			X					
		2h	10:00	OGTT	X	X					
		2h 15m	10:15		X						
		2h 30m	10:30		X	X					
		2h 45m	10:45		X						
		3h	11:00		X	X				X	
		3h 30m	11:30		X						
		4h	12:00		X	X					
		4h 30m	12:30		X						
		5h	13:00	점심식사 (OGTT 후) (Lunch after OGTT)	X						
		6h	14:00			X			6~12h	X	
		8h	16:00			X				X	
		10h	18:00			X					
		12h	20:00	퇴원 7) (Discharge[7)])		X				X	

1) 약동학: D2 Metformin 투여 전 및 투여 후 0.5, 1, 1.5, 2, 2.5, 3, 4, 6, 8, 10, 12시간
   약력학: 약물 투여 후 2시간 이후 OGTT(75g glucose) 투여 전 및 투여 후 15분, 30분, 45분, 60분, 90분, 120분, 150분, 180분
2) 유전형 검사는 3mL 채혈하며 1D 1.5h, 대사체 검사: 채혈(5mL), 채뇨(10mL)
3) 250 mg용량군: Metformin 375 mg(1D 12h), Metformin 250 mg (2d 0h)
   1000 mg: Metformin 1000 mg(1D 12h), Metformin 1000 mg (2d 0h)
4) 약동학 집뇨: Metformin 복용 전 방광을 비우고 복용 후 다음의 시간 별 집뇨를 실시한다. → 0-6시간, 6-12시간
5) 안전성 검사: 스크리닝, 1D, 2D에 시행.
   스크리닝: 심전도, 혈액학, 임상화학, 소변, vital sign, 신체검사
   1D: 혈액학, 임상화학, 소변
   2D: 임상화학(LDH 만 시행), vital sign
6) 이상 반응 발생, 병용 약물 사용 여부는 1D 입원 시부터 2D 퇴원 시까지 지속적으로 확인한다.
7) -1D 오후 5시경 입원 후 6시경 저녁식사, D2 오후 8시경 퇴원

목 차 (Table of contents)

1 연구의 명칭 및 실시기관 (TITLE AND STUDY CENTER) 3
2 임상연구 담당자 (INVESTIGATORS) 3
3 임상연구의 목적 및 배경 (PURPOSE AND BACKGROUND) 3
3.1 배경 (BACKGROUND) 3
3.2 연구목적 (PURPOSE OF THE STUDY) 4
4 윤리적 고려사항 (ETHICAL CONSIDERATIONS) 4
5 임상연구용 의약품 (STUDY DRUG) 4
5.1 연구약 (STUDY DRUG) 4
5.1.1 Metformin 4
5.2 적응증 (INDICATION) 4
5.3 용법, 용량, 예측 이상반응(부작용), 상호작용 및 사용상의 주의사항 (DOSE, USAGE, ADVERSE EVENTS, DRUG INTERACTION, AND CAUTIONS) 4
5.4 임상연구용의약품의 공급 및 라벨링 (LABELING OF STUDY DRUG) 5
5.5 임상연구용의약품의 관리 (STUDY DRUG MANAGEMENT) 5
6 연구대상자 선정기준 및 연구대상자 수 산출 근거 (SUBJECT ELIGIBILITY AND DETERMINATION OF SUBJECT NUMBER) 5
6.1 포함기준 (INCLUSION CRITERIA) 5
6.2 제외기준 (EXCLUSION CRITERIA) 5
6.3 연구대상자 수 (NUMBER OF SUBJECTS) 6
7 연구대상자의 관리 (SUBJECT MANAGEMENT) 6
8 임상연구 기간 (STUDY PERIOD) 6
9 임상연구 방법 (STUDY METHODS) 6
9.1 연구대상자 군 및 개요 (SUMMARY OF STUDY) 7
9.2 투약 방법 및 임상연구 (STUDY DRUG ADMINISTRATION) 7
9.3 채혈방법, 채혈량 및 검체보관 (SAMPLING METHOD) 7
9.4 순응도 평가 (ASSESSMENT OF COMPLIANCE) 8
9.5 채혈시간의 설정 (SAMPLING TIME POINT) 8
9.6 연구대상자 SN 및 AN 부여 방법 (ALLOCATION OF SUBJECT NUMBER) 8
9.7 연구대상자 제한사항 (RESTRICTIONS TO SUBJECT) 9
9.8 관찰 및 검사 항목 (OBSERVATIONS) 9

9.8.1 스크리닝 검사(D-30~D-1) (SCREENING) 9
9.8.2 투약 전/후 검사 (TREATMENT PERIOD) 9
9.8.3 약물유전학 검사 (GENOTYPING) 9
10 연구대상자의 대체 및 탈락 기준 (DROPOUT AND REPLACEMENT) 10
10.1 연구대상자의 탈락 (DROPOUT) 10
10.2 연구대상자의 대체 (REPLACEMENT) 10
10.3 연구대상자의 탈락 기준 (DROPOUT CRITERIA) 10
11 연구대상자의 안전 보호 및 이상반응 발생 등에 대한 대책 (SAFETY CONCERNS AND PLAN FOR ADVERSE EVENTS) 10
12 평가방법 (ASSESSMENTS) 11
12.1 약동학 및 약력학적 평가 (PHARMACOKINETIC/PHARMACODYNAMICS) 11
12.2 통계분석 (STATISTICAL ANALYSIS) 11
12.3 안전성 평가 (SAFETY ASSESSMENTS) 11
13 이상반응 (ADVERSE EVENT, AE) 12
13.1 이상반응의 정의 (DEFINITION OF ADVERSE EVENT) 12
13.2 이상약물반응의 정의 (DEFINITION OF ADVERSE DRUG REACTION) 12
13.4 이상반응의 기록 (RECORDING OF AE) 12
13.5 이상반응의 중증도 및 연구약과의 인과관계 평가 (AE SEVERITY AND RELATIONSHIP WITH STUDY DRUG) 13
13.5.1 이상반응의 중증도 평가 (AE SEVERITY) 13
13.5.2 이상반응의 연구약과의 인과관계 평가 (RELATIONSHIP WITH STUDY DRUG) 13
13.5.3 이상반응의 추적 관찰 (AE FOLLOW-UP) 13
13.6 이상반응(부작용) 보고 (REPORTING OR AE) 14
13.6.1 중대한 이상반응의 보고 (REPORTING SERIOUS AE) 14
14 연구대상자 설명문 및 동의서 양식 (INFORMED CONSENT) 14
15 관련자료 및 문서보관 (STORAGE OF DOCUMENTS) 14
16 비밀보장 (CONFIDENTIALITY) 14
17 임상연구자료의 신뢰성보증 (QUALITY ASSURANCE) 14
18 자료 안전 모니터링 계획 (DATA SAFETY MONITORING PLAN) 15
19 참고문헌 (REFERENCES) 15
[별지 1] 용법, 용량, 예측 이상반응(부작용), 상호작용 및 사용상의 주의사항
([APPENDIX 1] DOSE, USAGE, ADVERSE EVENTS, DRUG INTERACTION, AND CAUTIONS)

1 연구의 명칭 및 실시기관

연구제목: 건강한 남성에서 Metformin 250/1000 mg 투여 시 약동-약력학 특성을 평가하기 위한 연구

An open-label, parallel group clinical trial to investigate the pharmacokinetics/pharmacodynamics of 250/1000 mg of metformin IR after oral administration in healthy male volunteers

실시기관: 분당서울대학교병원

2 임상연구 담당자

연구책임자:

분당서울대학교병원 교수 정재용

연구담당자:

서울대학교병원 임상약리학과 전공의	김언혜, 오재성, 장경호, 문설주, 박상인, 윤장수, 정혜원
분당서울대병원 임상시험센터 간호사	김성희, 심덕선, 이은경
분당서울대병원 임상시험센터 연구원	조정희, 이은아, 박세나

관리약사:

분당서울대병원 임상시험센터 약사 오유미, 강선미

3 임상연구의 목적 및 배경

3.1 배경

당뇨병 환자의 90%이상을 차지하는 제 2 형 당뇨의 일차치료제로 널리 사용되는 경구 혈당강하제인 메트포민(metformin)은 hepatocyte 등의 작용부위에서 세포내로 이동하기 위해 양이온수송단백(Organic Cation Transporter, OCT)을 통해 수송되며(1) 이의 유전자형의 변이가 메트포민의 효과에 미치는 것으로 보고되어 있다(2).

이전 연구결과에 따르면, 일본인 제2형 당뇨병 환자를 대상으로 저용량 (500 to 750 mg daily) metformin을 6개월간 투여한 경우, 공복혈당의 경우 18-23%, HbA1c의 경우, 11-15% 감소효과를 나타내었다고 한다(3). 또한 저용량의 metformin은 젖산 농도의 최대치를 상승시키는 것을 막음으로서 젖산산증 발생 가능성을 낮출수 있다(4). 뿐만 아니라 metformin의 대표적인 부작용으로 알려져 있는 소화기계 부작용 발생빈도는 용량과 비례적인 관계를 나타낸다는 연구결과가 있어 이러한 부작용을 줄일 수 있다는 장점이 있다(5). 그러나 저용량에서의 metformin의 약동/약력학적인 작용에 대해서는 연구된 바가 적으며, 특히 500 mg 이하의 용량에 대해서는 탐색되어 있지

않아, 이번 연구에서는 저용량에서의 metformin의 약동/약력학적 특성을 고용량에서와 비교, 탐색하고자 한다. 또한, 개인의 유전자 형에 따라서 metformin의 약동/약력학적 특성이 어떻게 다른지에 대해서도 함께 연구해보고자 한다.

3.2 연구목적

① 건강 성인 남성을 대상으로 저용량 및 고용량 메트포민 투여 시의 약동학 및 약력학(혈당강하효과)을 비교 평가한다.

② 건강 성인 남성을 대상으로 저용량 및 고용량 메트포민 투여 시의 유전형과 내인성 대사체의 변화에 대해 탐구한다.

4 윤리적 고려사항

본 연구는 분당서울대학교병원 생명윤리심의위원회(IRB)의 승인과 헬싱키 선언 및 약사법 시행규칙(보건복지부령 제 52호, 2011.5.6) 별표 3의 2 등 관련규정을 준수하여 실시할 것이다. 이에 따라 연구의 목적 및 임상연구 의약품의 특성을 연구대상자에게 설명문을 통해 설명하며, 연구의 목적 및 위험 등을 알고 동의서를 작성한 자원자만을 연구에 참여 시킬 것이며, 연구대상자는 연구기간 중 언제라도 본인이 원할 경우 연구 참여 동의를 철회할 수 있다는 것도 설명할 것이다. 연구기간 동안 얻어지는 결과들은 증례기록서에 기록될 것이며 모든 사항에 대해서는 비밀을 보장할 것이다. 연구대상자 보상에 관한 사항도 연구대상자에게 설명할 것이다.

5 임상연구용 의약품

5.1 연구약

5.1.1 Metformin

① 제품명: 다이아벡스정 1000 mg, 250 mg (대웅제약)

② 제형 및 성상: 백색의 원형 필름 코팅정

③ 함량: metformin HCL 1000 mg, 250mg

5.2 적응증

- 다이아벡스정: 제2형 당뇨병 환자(인슐린 비의존형)의 혈당조절을 위하여 식사요법 및 운동요법과 함께 보조치료제로 사용

5.3 용법, 용량, 예측 이상반응(부작용), 상호작용 및 사용상의 주의사항

<별첨1> 참조

5.4 임상연구용의약품의 공급 및 라벨링

임상연구에 사용되는 의약품은 임상연구책임자가 연구약의 제조회사 등에 의뢰하여 공급을 받은 후, 임상시험센터 관리약사에게 공급한다.

연구약은 1 회 투약 분량이 개별 포장된다. 임상시험용의약품 라벨에는 약사법시행규칙 제 75 조제 6 법에 따라, 아래의 사항을 기재한다.

1. "임상시험용"이라는 표시
2. 제품의 코드명 또는 주성분의 일반명
3. 제조번호 및 사용(유효)기간 또는 재검사일자
4. 저장방법
5. 임상시험계획 승인을 받은 자의 상호와 주소
6. "임상시험외 목적으로 사용할 수 없음"이라는 표시

5.5 임상연구용의약품의 관리

임상연구용의약품 관리약사 (이하 '관리약사')는 임상연구에 사용되는 의약품의 인수, 보관, 관리 및 반납에 대한 책임을 갖는다. 약사법 시행규칙 (보건복지부령 제 52호, 2011. 5. 6) 별표 3의 2의 규정에 따라 관리약사는 연구용의약품의 수령사실 및 수량을 확인하고 서명해야 하며, 적절히 관리한다. 임상연구용의약품이 임상연구계획서에 따라서 연구대상자에게 투여되도록 하고, 각 연구대상자에게 지급된 임상연구용의약품 및 관리에 대한 기록을 정확히 한다. 사용되지 않은 임상연구용의약품은 파기가 결정할 때까지 보관한다.

.

6 연구대상자 선정기준 및 연구대상자 수 산출 근거

6.1 포함기준

1) 한국인 성인으로 만 20~45세, 체중 50~100 kg 이며, Ideal Body Weight ((Height – 100) X 0.9)의 25 % 이내인 남성
2) 스크리닝 검사시 시행한 공복 혈당 검사(FPG)에서 70-125 mg/dL 의 혈당 범위를 보이는 자
3) 신뢰할 수 있고, 임상연구 기간 동안 시간을 할애하고 임상연구 계획서를 준수할 의지가 있는 연구대상자
4) 임상연구에 대한 자세한 설명을 듣고 완전히 이해한 후, 자의로 참여를 결정하고 주의사항을 준수하기로 서면 동의한 자

6.2 제외기준

1) 임상적으로 유의한 호흡기, 순환기, 신장, 위장관, 간, 내분비, 혈액, 신경 (운동실조 포함),

정신 질환 혹은 기타 만성 질환, 알코올 혹은 약물 중독에 대한 임상적인 증거가 있는 자

2) 연구약의 흡수에 영향을 줄 수 있는 위장관계 질환(크론씨병, 궤양, 급성 또는 만성 췌장염 등) 이나 위장관계 수술(단, 단순 맹장수술이나 탈장수술은 제외) 의 과거력이 있는 자
3) 임상연구용 의약품의 투약 14일 이내에 유의한 약물상호작용이 알려진 약물과 임상 연구자가 판단하기에 적합하지 않다고 판단되는 약물을 복용한 자.
4) 임상연구 개시 전 30일 이내 혈액을 공여하거나 60일 이내에 다른 임상연구용의약품 혹은 시판 중인 약물의 임상연구에 참여한 자
5) 임상연구용 의약품 및 동일계열의 성분을 포함한 약물 및 기타 다른 약물(아스피린, 항생제 등) 에 대한 과민반응 또는 임상적으로 유의한 과민반응의 병력이 있는 자
6) 연구기간 중 임신을 계획하고 있거나 계획 중이 아니어도 인정받는 피임법 (예: 본인 및 파트너의 불임수술, 파트너의 자궁 내 피임기구, 간벽 피임법, 격막 또는 콘돔의 병용)을 사용할 수 없는 자
7) 지속적으로 음주(21 units/week 초과, 1 unit = 10 g of pure alcohol)를 하거나 임상연구기간 중 금주할 수 없는 자
8) 임상실험실검사 결과를 비롯한 기타 사유로 인하여 연구자가 임상연구 참여에 부적합하다고 판단한 자

6.3 연구대상자 수

본 연구는 탐색적 연구로서 연구대상자 산출을 위한 통계적 분석을 적용하지 않으며, 연구 목적을 충족시키는 한 가능한 최소의 연구대상자를 대상으로 하는 것이 바람직하다. 이와 같은 연구를 교차연구로 설계 했을 때 각 용량군당 10 명의 연구대상자가 적당하며, 이번 연구는 총 2용량군, 20명의 연구대상자를 대상으로 진행한다.

7 연구대상자의 관리

지원자에게 투약 7일 전부터 연구 종료 시까지 과도한 운동, 음주 (알코올 > 30 g)나 흡연 (> 10 개피)을 하지 않고 투약 14일 전부터 일체의 타 약물의 복용을 하지 않도록 주의시킨다.

8 임상연구 기간

IRB 승인일 이후 1년 또는 총 20명의 연구대상자가 임상연구를 완료하는 시점으로, 각 연구대상자는 입원 후 이틀 뒤에 임상연구를 종료함.

9 임상연구 방법

9.1 연구대상자 군 및 개요

250 mg 용량군과 1000 mg 용량군의 각 용량군별 10명씩 총 20 명의 연구대상자를 대상으로 투약한다.

9.2 투약 방법 및 임상연구

모든 투약은 임상연구담당자의 감독하에 실시된다
연구대상자 전원은 연구시작 1주일 전부터 상담을 거쳐 최소 200-250g/day의 carbohydrate의 식사를 하며 이는 연구 시작 3일 전부터는 철저히 지켜져야 한다.
선정된 연구대상자는 첫 번째 입원 예정일 (-1d) 오후 5시까지 분당서울대병원 임상시험센터에 입원한다. 입원 후 저녁 6시경 임상시험용식사를 한다. 입원 둘째 날 (1d) 오전 기상 후 첫 소변을 수집하며 8시경 유전형 및 대사체 채혈, 안전성 검사를 시행한다. 오전 10시경부터 경구 당부하 후 약력학 채혈을 시작하며 마지막 약력학 채혈(오후 1시경) 후 점심식사를 제공한다. 오후 6시경 저녁식사를 제공하며, 오후 8시경 각 용량군 별로 metformin 375 mg 또는 1000 mg을 240 mL의 물과 함께 복용한다. 셋째 날 (2d) 오전 기상 후 첫 소변을 수집한다. 오전 8시경 대사체 채혈 및 투약 전 약동학 채혈을 수행 후 각 용량군 별로 metformin 250 mg 또는 1000 mg을 공복 상태에서 240 mL의 물과 함께 복용한다. 투약 직전 방광을 비운 후 12시간 동안 집뇨를 수행한다. 이후 예정대로 약동학 채혈을 수행하며, 2시간 뒤 오전 10시경부터 경구 당부하 후 약력학 채혈을 수행한다. 예정된 약동학 채혈을 모두 수행 후 연구대상자는 오후 8시경 퇴원할 수 있다. 퇴원시 안전성 검사에 해당하는 검사들을 시행하며 이 검사들에서 특별한 이상이 발견되지 않는 경우 임상연구를 종료한다.

9.3 채혈방법, 채혈량 및 검체보관

약동학 평가를 위해 7ml, 약력학 평가를 위해 2 mL씩 각각 채혈한다.
또한, 입원 채혈 시 반복적 채혈을 위해 연구대상자의 팔 또는 손등의 정맥부위에 heparin-locked catheter를 설치하고. 채혈 시 채혈세트 안에 남아 있는 생리식염수를 제거하기 위해 매번 약 0.5 mL의 혈액을 빼내어 버린 후 혈액을 채취하고, 다시 catheter 안에 잔류하는 혈액의 응고를 방지하기 위하여 넣은 주사용 생리 식염수 1 mL를 주입한다.
채혈된 혈액을 헤파린이 포함된 채혈용기 (tube) 에 취한 후 약동학 평가를 위한 혈액은 채혈 후 30분 이내에 3000 rpm 으로 10분 동안 원심분리한 후, 상층액의 혈장만을 분리하여 분석시까지 -20℃이하에서 보관한다. 약력학 평가를 위한 혈액은 즉시 진단검사의학과에 접수하여 분석을 바로 실시 한다.
각 tube 에는 연구명, 연구대상자번호(AN), 채취시간 (약물 투여시간 기준), 채취일 등을 기록한 라벨을 부착한다.

9.4 순응도 평가

입원시 투약이 있는 날에는 연구 담당자가 직접 투약을 하며 복용을 확인한다.

9.5 채혈시간의 설정

[약동학 채혈]
약동학적 평가를 위한 채혈시간은 D2에 2번째 Metformin을 투여한 직전과 후 0.5, 1, 1.5, 2, 2.5, 3, 4, 6, 8, 10, 12시간 채혈을 실시한다.

[약력학 채혈]
약력학 채혈은 혈당강하 효과를 보기 위한 것으로 OGTT직전 과 후 15, 30, 45, 60, 90, 120, 150, 180분에 채혈을 실시한다. 일정은 일정표를 참조로 한다.

약동학 및 약력학 채혈 일정은 기존의 보고가 많으며 참고문헌(2,6)을 근거로 설정하였다.

[안전성 검사]
안전성평가를 위한 채혈은 스크리닝 시와 연구 개시 및 연구 종료 시(D1, D2)에 실시한다.

9.6 연구대상자 SN 및 AN 부여 방법

서면 동의를 받은 순서대로 자원자에게 스크리닝 번호(SN: Screening number)를 부여한다. 스크리닝 번호는 01로 시작하는 전체 숫자 두 자리로 구성된다. 재검까지 고려하여 최종적으로 스크리닝 통과가 결정되면 배정번호(AN: allocation number)를 부여한다. 본 임상연구에서 각 용량단계로의 배정은 open label로서, 연구자 또는 연구대상자는 어느 용량단계에 배정되었는지 알 수도 있다.

배정보호는 총 3자리 숫자이며, 다음 두 가지로 구성된다.

- ■ 용량군 번호
  (저용량군 (375/250 mg): 1; 고용량군(1000/1000 mg): 2)
- ■ 각 연구기관에서 순차적으로 부여된 두 자리의 연구대상자 번호

예를 들어 ‘AN105’는 250 mg 용량군(375/250 mg)의 다섯번째 연구대상자를 의미하며 ‘AN203’은 1000 mg 용량군(1000/1000 mg)의 세번째 연구대상자를 의미한다. 각 연구대상자에게 부여된 연구대상자번호는 임상연구가 끝날 때까지 연구대상자를 인식하는 연구대상자식별코드(subject identification code)로 사용된다. AN 배정 후 연구대상자가 투약 전 동의철회나 다른 이유로 인해 임상연구에 참여하지 못하는 경우 예비연구대상자를 두어 대체하는 것으로 한다. 투약 전 중도 탈락 한 대체 연구대상자의 AN 번호는 탈락한 연구대상자의 AN 번호를 그대로 사용하나 투약

후 탈락한 경우 대체 연구대상자의 AN번호는 해당 용량군의 마지막 연구대상자번호 이후의 번호를 새로 부여한다.

9.7 연구대상자 제한사항

- 임상병리검사 (혈액학검사, 혈액화학검사, 뇨검사) 10시간 전부터 금식한다. 투약 7일 전부터 임상연구가 종료되는 시점까지 비정상적인 식사나 과도한 운동을 해서는 안된다.
- 연구대상자는 투약 기간 동안 알코올을 제한하며, 입원전 3일(72시간) 전부터 퇴원 후 48시간 까지 알코올을 섭취할 수 없다. 활력징후 측정 전 30분부터는 금연하여야 한다.
- 연구대상자는 투약 14일 전부터 임상연구 종료시까지 약물상호작용이 알려진 약물을 복용할 수 없다.

9.8 관찰 및 검사 항목

9.8.1 스크리닝(Screening) 검사(D-30~D-1)

다음과 같은 항목으로 실시한다.
문진, 신체검사, 임상병리(혈액학, 혈액화학, 뇨) 검사, 심전도, vital sign
- 혈액학 검사: WBC, RBC, Hemoglobin, Hematocrit, Platelet, differential count of WBC(Neutrophil, Lymphocyte, Monocyte, Eosinophil, Basophil)
- 혈액화학적 검사: Fasting Glucose, Creatinine, Albumin, Total Bilirubin, SGOT, SGPT
- 뇨검사: Specific Gravity, pH, Protein(albumin), Glucose, Ketone, Bilirubin, Blood, Nitrite, WBC,

Urobilinogen, Microscopy

9.8.2 투약 전/후 검사

- 약력학 검사: 혈중 glucose level을 검사하는 것으로 D1, D2 에 실시
- 안전성 검사: D1, D2 (혈액화학적 검사 중 LDH 만 시행)
- 혈액학 검사: WBC, RBC, Hemoglobin, Hematocrit, Platelet, differential count of WBC(Neutrophil, Lymphocyte, Monocyte, Eosinophil, Basophil)
- 혈액화학적 검사: Creatinine, Albumin, Total Bilirubin, SGOT, SGPT, LDH
- Vital sign: D2 8h
- 대사체 검사: D1, D2 에 실시 (혈액, 소변)

9.8.3 약물유전학 검사

- 약물반응과 관련된 유전자를 탐색하기 위해 등록된 연구대상자를 대상으로 서면동의 후 유전자 검사를 실시
- 검사대상 유전자: 메트포민의 약물반응에 관여하는 것으로서 OCT1, OCT2, OCT3, MATE1,

MATE2-K, PMAT, OAT, MDR, MRP, BCRP등의 약물 수송체 유전자형
- 검사일정: D1 투약 전 안전성 검사 시 1회 3ml 채혈

10 연구대상자의 대체 및 탈락 기준

10.1 연구대상자의 탈락

투약 및 계획된 약동학 및 약력학적 평가를 위한 채혈을 모두 마친 연구대상자를 연구의 완료자로 한다. 약동학 및 약력학적 평가를 위한 채혈이 완전히 끝나지 않았거나 연구기간 중 다른 약물을 병용하는 등 유의한 계획서 이탈이 있는 경우 연구담당자의 판단에 따라 중도 탈락자로 한다.

10.2 연구대상자의 대체

연구를 중지하거나 연구에서 탈락되는 경우 새로운 연구대상자로 대체할 수 있다. 그러나 중지 시점까지 해당 연구대상자로부터 얻은 연구결과는 최종 평가 시 평가 가능한 항목에 대해서 검토될 수 있다.

10.3 연구대상자의 탈락 기준

연구대상자의 연구탈락은 연구기간 중 어느 시점에서나 판정가능하며, 연구대상자 자의에 의해서 연구에서 탈락하는 경우에 그 사유는 불문하나 탈락 시에는 연구담당자 또는 책임자에게 보고토록 한다. 임상연구자는 다음과 같은 경우에 연구대상자를 연구에서 탈락시킬 수 있다.

- 연구대상자가 임상연구 중 연구약의 투여중단을 요구하거나, 연구참여 동의를 철회한 경우
- 중대한 이상반응/이상약물반응이 나타나는 경우
- 연구대상자가 이상반응이 발생하여 연구약의 복용을 거부하거나 더 이상 연구약의 복용이 힘들다고 판단되는 경우
- 연구대상자가 연구약의 안전성을 평가하는데 영향을 줄 것으로 예상되는 약물을 임의로 복용한 경우
- 연구자의 판단으로 환자를 위해 연구의 중지가 필요하다고 생각되는 경우
- 연구대상자가 제때에 방문을 안 하며 연락이 안 되는 경우

11 연구대상자의 안전 보호 및 이상반응 발생 등에 대한 대책

- 임상연구 책임자 및 연구담당자는 연구계획을 정확히 분석, 숙지하여 예기치 않은 이상

반응 출현에 대해 충분한 대처와 필요에 따른 보고, 참여 연구진에 대한 교육 등 사전 조치를 취한다. 임상연구의 진행은 의약품임상시험관리기준 (KGCP)에 합당하게 진행한다.

- 본 연구 도중 부작용이 발생하는 경우에는 적절한 조치를 취한다. 즉, 중대하고 예기치 못한 이상약물반응의 발생시 IRB에 보고한다. 이상반응 등에 대한 필요한 검사 및 치료는 의학적으로 관행적인 요법에 준하여 시행한다.
- 응급상황 발생시 분당서울대학교병원 및 임상시험센터 응급상황에 대한 대처방안에 준하여 조처한다.

12 평가방법

약동학 및 약력학적 평가와 통계분석은 모든 채혈 일정을 완료한 자를 대상으로 한다. 안전성 평가는 한번이라도 투약 받은 연구대상자를 대상으로 한다.

12.1 약동학 및 약력학적 평가

[약동학 지표]: AUC, C_{max}, $t_{1/2}$, CL_R, Fe 및 약동학 구획 모델의 속도상수
[약력학 지표]: 혈당농도곡선하면적 (AUGC), 최대혈당치(G_{max})
투약 전후 내인성 대사체(steroid profile 등)

위의 지표들에 대하여 비구획적 방법과 집단 비선형혼합효과 모델링(NONMEM)을 이용하여 약동학/약력학 파라미터 산출하고, 산출된 파라미터에 대한 유전형별 공변량 효과 분석 및 정량화를 실시한다.

12.2 통계분석

약동/약력학적 파라미터를 기술통계적으로 분석하며, 파라미터의 성질에 따라 실측치 또는 로그 변환한 값으로 군간 비교를 실시한다. 이 때 모수적 또는 비모수적 방법으로 분석한다.
또한 인구학적 정보는 약동학 및 약력학 평가에 있어서 집단 비선형혼합효과 모델링의 정보로 이용될 수 있다. 모든 분석에서 $P < 0.05$이면 통계적으로 유의한 것으로 판정한다.

12.3 안전성 평가

1) 임상검사
각 임상검사치의 정상, 이상은 개별 연구대상자에서 판정하며, 필요한 경우 통계적 검증을 할 수 있다. 임상검사치 이상에 대하여 그 정도를 기재하고 연구약과의 관련성을 판정한다.
2) 모든 연구대상자의 임상검사치를 도표화하여 총괄적으로 검토한다.

13 이상반응

13.1 이상반응(Adverse Event, AE)의 정의

임상연구에 사용되는 의약품을 투여 받은 연구대상자에서 발생한, 바람직하지 않고 의도되지 않은 증후(sign, 예: 실험실적 검사치의 이상), 증상(symptom), 질병을 말하며, 해당 임상연구에 사용된 의약품과 반드시 인과관계를 가져야 하는 것은 아니다.

13.2 이상약물반응(Adverse Drug Reaction, ADR)의 정의

임상연구에 사용되는 의약품의 임의의 용량에서 발생한, 모든 유해하고 의도되지 않은 반응으로서, 임상연구에 사용되는 의약품과의 인과관계를 배제할 수 없는 경우이다.

13.3 중대한 이상반응/이상약물반응(Serious AE/ADR)의 정의

임상연구에 사용되는 의약품의 임의의 용량에서 발생한 이상반응 또는 이상약물반응 중에서 다음과 같은 경우를 말한다.

- 사망을 초래하거나 생명을 위협하는 경우
- 입원 또는 입원기간의 연장이 필요한 경우
- 지속적 또는 의미 있는 불구나 기능 저하를 초래하는 경우
- 선천적 기형 또는 이상을 초래하는 경우
- 기타 의학적으로 중요한 상황

13.4 이상반응의 기록

이상반응은 의학 진단 용어로 기록하여야 하며, 이것이 불가능할 경우 임상연구책임자 또는 담당자가 관찰하거나, 연구대상자가 보고한 증상 및 징후에 대한 용어를 기록하여야 한다.

연구 시작 전에 연구대상자에게 나타나는 증상 및 징후는 연구대상자의 증례기록서에 기록하여야 한다. 동의서 취득 후, 임상연구용 의약품의 투약 전에 나타난 이상반응은 Screening, Pre-dose 문진 및 이학검사 기록지에 기록하여야 한다. 연구약 투약 후 나타난 모든 이상반응은 약물과 인과관계가 없더라도 CRF의 이상반응 기록지에 기록하여야 한다.

임상연구 도중 또는 최종투약 후 30일 이내에 나타나는 중대한 이상반응은 연구약과 인과관계가 없더라도 생명윤리심의위원회에 관련규정에 따라 보고하여야 한다. 이상반응은 연구책임자 또는 담당자가 평가한다. 처음 기록되는 이상반응은 CRF의 이상반응 모니터링 기록서에 기록한다. CRF에는 이상반응의 증상 및 증후, 연구약과 관련하여 취해진 조치, 발현 날짜 및 시간(가능한 경우), 심각한 정도(최대 강도; maximal intensity), 경과(course; 즉, 지속적 또는 간헐적), 이상반응의 심각성 또는 경중(seriousness), 결과(outcome), 연구약과의 인과관계를 기재하여야 한다. 세부적인

용량 변경 및 치료 내용은 CRF의 적절한 page에 기록하여야 한다.

이전에 CRF에 기록된 적이 있고, 결과(outcome) 항에 '진행 중'이라고 표시한 이상반응은 필요한 경우 이후 방문에서 검토되어야 한다. 이상반응이 회복된 경우에는 CRF의 기록이 완성되어야 한다. 만약 연구 기간 중에 이상반응의 빈도 및 심각성이 증가한다면, 새롭게 이상반응 기록서에 기록을 시작한다.

이상반응을 알아내는 한 가지 일관된 방법으로서 연구대상자의 자발적인 보고 외에도, 투약시마다 연구대상자에게 다음과 같이 원하는 대답을 유도하지 않는 방식으로 질문을 한다. "지난 방문 또는 투약 이후로 다르게 느껴진 바는 없습니까?"

13.5 이상반응의 중증도 및 연구약과의 인과관계 평가

13.5.1 이상반응의 중증도 평가

이상반응의 중증도는 최대 강도(maximal intensity)에 의거하여 아래의 기준에 의해 분류한다.

1) 경증 (mild): 연구대상자의 정상적인 일상생활(기능)을 저해치 않고, 최소한의 불편을 야기하여, 연구대상자가 쉽게 견딜 수 있는 경우
2) 중등도 (moderate): 연구대상자의 정상적인 일상생활(기능)을 유의하게 저해하는 불편을 야기하는 경우
3) 중증 (severe): 연구대상자의 정상적인 일상생활(기능)을 불가능하게 하는 경우

13.5.2 이상반응의 연구약과의 인과관계 평가

임상연구용 의약품과의 인과관계는 다음과 같이 6단계로 분류하고, 연구담당자의 견해를 부가한다.

1) 명확히 관련성 있음 (Definitely related)
2) 관련성이 있다고 생각됨 (Probably related)
3) 관련 가능성이 있음 (Possibly related)
4) 관련성이 없다고 생각됨 (Probably not related)
5) 명확히 관련성 없음 (Definitely not related)
6) 알 수 없음 (Unknown)

13.5.3 이상반응의 추적 관찰

임상연구 책임자 또는 담당자는 이상반응이 나타난 연구대상자에 대해 증상이 가라앉고 비정상적 임상검사치가 기준치로 회복되거나, 혹은 관찰된 변화에 대해 만족스러운 설명이 될 때까지 추적 관찰하여야 한다.

13.6 이상반응(부작용) 보고

13.6.1 중대한 이상반응의 보고

중대한 이상반응이 임상연구용의약품 투여기간 도중 또는 최종 투약 후 30 일 이내에 발생하는 경우, 이상반응의 연구약과의 관련 여부와 관계없이 임상연구책임자 또는 담당자는 24시간 이내에 IRB에 보고하여야 한다.

14 연구대상자 설명문 및 동의서 양식

임상연구책임자/담당자는 본 연구의 실시에 있어 연구대상자 (또는 연구대상자의 법적 대리인) 에게 연구내용 및 연구약제의 효과, 이상반응에 대해 사전에 충분히 설명하고 연구대상자로부터 서면동의를 받아야 한다. 연구자는 서명된 동의서 원본은 연구 기록으로 보관하며, 서명된 동의서 사본 및 설명한 설명문은 연구대상자(또는 연구대상자의 법적대리인)에게 제공되어야 한다. <별첨> 연구대상자 동의 설명서 및 <별첨> 동의서 참조

15 관련자료 및 문서보관

임상연구 관련 문서에는 모든 작업일지, 근거문서(Source document), 모니터링 기록과 약속 일정, 임상연구 연구자와 관련 기관간의 서신교환과 규정문서 등이 포함된다(예: 연구자가 서명한 임상연구 계획서와 그 개정판, 생명윤리심의위원회 관련 서신, 허가관련서류, 허가/서명된 연구대상자 동의서, 임상약물 수령증, 수불기록).

근거문서는 모든 관찰기록과 임상활동에 관한 기록, 임상연구의 평가와 재구성에 필요한 모든 보고서와 기록을 포함한다. 따라서 근거문서에는 임상연구 계획서에 근거하여 실시된 모든 처치에 대한 기록 또는 이와 유사한 기록이 모두 포함된다.

관찰기록은 가능한 한 그 원본을 근거문서로서 보관해야 한다. 그러나 복사본이 원본과 정확히 동일하고 깨끗하며 읽기 쉽도록 기록되어 있으면 복사본도 근거문서로 간주할 수 있다.

16 비밀보장

연구대상자의 신원에 대한 모든 기록은 비밀보장이 되도록 주의한다. 다만, 점검을 실시하는 자, 연구심의위원회는 연구대상자의 비밀보장을 침해하지 않고 관련 규정이 정하는 범위 안에서 연구절차와 자료의 신뢰성을 검증하기 위해 연구대상자에 대한 기록을 열람할 수 있다.

17 임상연구자료의 신뢰성보증

임상연구 기간 동안 임상연구 계획서가 준수되고 있는지를 확인하고, 증례기록서를 각 연구대상

자의 근거문서와 비교하며, 임상연구에 사용되는 의약품의 수불기록을 확인하고 임상연구가 관련 규정에 따라 시행되고 있음을 확인한다. 연구대상자의 의무기록은 연구대상자 신분이 노출되지 않도록 기밀을 유지하며 검토된다. 연구 자료 및 기타 임상연구기본문서는 연구 종료 후 적어도 5년이 경과할 때까지 보관되어야 한다.

18 자료 안전 모니터링 계획

본 연구는 안전성이 검증된 시판된 약물 성분의 허가 사항내 용법에서 각각의 성분의 약물을 비교적 단기간 투여하여 약물상호작용과 유전자 형에 따른 약물 반응을 평가하기 위한 임상연구로 용량증량연구나 중추적(Pivotal) 연구가 아니므로 중간분석은 계획되지 않았다.

그러나 계획서의 연구대상자 모집 및 적합성, 복약 순응, 임상연구계획서 위반 및 중도하차, 데이터의 완전성 등에 대한 연구수행모니터링은 위험수준 Level 2에 준하여 시행할 것이다. 안전성 모니터링은 본 연구의 위험수준에 의거하여 Independent safety manager, 임상연구책임자가 보고한 이상반응에 대한 소견을 검토한다. 자료 안전 모니터링은 연구책임자가 수집된 안전성 검사 결과를 토대로 자료 분석 및 안전성을 검토하여 평가하며, 필요 시 외부 전문가의 자문을 구한다. 본 연구계획서를 토대로 한 연구진행 과정에 대한 자료안전모니터링 시행 결과는 중간보고 또는 종료/결과보고시에 해당 IRB에 보고하도록 한다.

19 참고문헌

1. Pacanowski MA, Hopley CW, Aquilante CL. Interindividual variability in oral antidiabetic drug disposition and response: the role of drug transporter polymorphisms. Expert Opinion on Drug Metabolism and Toxicology. 2008;4(5):529-44.
2. Koepsell H, Lips K, Volk C. Polyspecific organic cation transporters: structure, function, physiological roles, and biopharmaceutical implications. Pharmaceutical Research. 2007;24(7):1227-
3. Ohmura, Chie, et al. Efficacy of low-dose metformin in Japanese patients with type 2 diabetes mellitus. Current therapeutic research 59.12 1998;889-895.
4. Gregorio, Franco, et al. "Low dose metformin in the treatment of type II non-insulin-dependent diabetes: clinical and metabolic evaluations. Acta diabetologia latina 27.2 1990; 139-155.
5. Garber, M. D., et al. Efficacy of Metformin in Type II Diabetes: Results of a Double-Blind, Placebo-controlled, Dose-Response Trialfn1fn1This work was supported by Bristol-Myers Squibb Company, Princeton, New Jersey." The American journal of medicine 103.6 1997; 491-497.
6. Shu Y, Sheardown SA, Brown C, Owen RP, Zhang S, Castro RA, et al. Effect of genetic variation in the organic cation transporter 1 (OCT1) on metformin action. Journal of Clinical Investigation. 2007;117(5):1422-31.

[별지 1] **용법, 용량, 예측 이상반응(부작용), 상호작용 및 사용상의 주의사항**

1.다이아벡스 정(메트포민)

1.1 용법 용량

성인

투여 용량은 개인별로 약물효과와 내약성을 근거로 결정되어야 하며, 1일 추천 최대 용량을 초과하지 않아야 한다. 이 약은 위장관계 이상반응을 감소시키고 적절한혈당조절에 필요한 최소용량을 확인하기 위하여 반드시 저용량으로 투여를 시작해야 하며, 1일 2 ~ 3회, 1회 500mg을 식사와 함께 투약한다. 용량의 증가는 매주 500mg씩 점차적으로 증량하며, 2000mg까지 투약할 경우는 보통 1일 2회 아침, 저녁으로 분할하여 투약한다. 2000mg을 초과하는 경우에는 1일 3회로 나누어 식사와 함께 투약하고 1일 최대용량은2500mg이다. 투약 개시 및 용량 조절 시 이 약에 대한치료반응을 측정하고 최소 유효용량을 확인하기 위하여 공복 시 혈당을 측정해야 한다. 혈중 당화 혈색소 농도를 약 3개월 간격으로 측정하여야 한다. 이 약의 치료 목표는 단일요법으로 투여하거나, 설폰요소계 또는 인슐린과 병용투여 시, 최소 유효용량으로 투여하여 공복 시 혈당과 혈중 당화 혈색소 농도를 정상 수준 혹은정상과 가까운 수준으로 감소시키는 것이다.

일반적으로 식사요법만으로 혈당이 조절되던 환자가 일시적으로 혈당이 조절되지 않을 경우, 이 약을 단기간 투여하는 것으로도 충분한 효과를 얻을 수 있다.

소아 및 성장기 청소년

이약은 10세 이상의 소아 또는 성장기 청소년에 사용할 수 있다. 일반적인 개시용량은 1일 1회 500 밀리그람이며 식사 중 또는 식사 종료 후 투여한다. 일주일 후에 혈당 수치를 측정하여 용량을 조절해야 한다. 위장관계 부작용은 점진적인 용량 증가로 개선될 수 있다. 1일 최대용량은 2000mg이며. 해당 투여량을 2~3회로 나누어 투약한다.

1.2 사용상의 주의사항

1. 경고

심한 유산산증 또는 저혈당증을 일으킬 수 있다.

2. 다음 환자에게는 투여하지 말 것.

1) 심혈관계 허탈(쇽), 급성심근경색과 패혈증과 같은 상태로부터 야기될 수 있는 신장질환이나 신기능부전(혈청크레아티닌치가 남자는 1.5mg/dL 이상, 여자는 1.4mg/dL 이상인, 또는 크레아티닌 청소율이 비정상인) 환자

2) 약물치료가 필요한 울혈성 심부전 환자

3) 방사선 요오드 조영물질을 정맥내 투여하는 검사(예 : 정맥요로조영술, 정맥담관조영술, 혈관조영술, 조영제를 사용한 컴퓨터단층촬영술 등)를 받는 환자(급성신부전을 일으킬 수 있고, 이 약을 투여 받는 환자에서는 유산산증과 관련이 있다. 따라서 이러한 검사가 계획된 환자에서는 이 약을 적어도 투여 48시간 전에 중지해야 하고, 48시간 이후에 신기능을 재평가하고 정상으로 판명된 이후에

만 치료를 재개한다.)
4) 이 약 또는 비구아니드계 약물에 과민반응의 병력이 있는 환자
5) 제 1형 당뇨병, 혼수를 수반하거나 그렇지 않은 당뇨병케톤산증을 포함하는 급성 또는 만성 대사성산증 환자 및 케톤산증의 병력이 있는 환자[제 1형 당뇨병과 당뇨병케톤산증은 인슐린으로 치료한다.]
6) 중증감염증 또는 중증 외상성 전신장애 환자에서는 이 약의 치료는 일시적으로 중지되어야 하고 환자의 경구적 섭취가 회복되고 신기능이 정상으로 판명될 때 치료를 다시 시작해야 한다. 수술 과정의 경우에(음식과 수액의 섭취에 제한이 없는 가벼운 수술은 제외) 이 약은 수술 48시간 전에 일시적으로 중지되어야 하고, 최소 48시간이 지난 후 신기능이 정상이라고 판명된 후에 치료를 다시 시작해야 한다.
7) 영양불량상태, 기아상태, 쇠약상태, 뇌하수체기능부전 또는 부신기능부전 환자
8) 간 기능장애(손상된 간 기능은 유산산증의 몇몇 경우와 관련이 있기 때문에, 일반적으로 임상적 또는 실험실적으로 간 질환의 증거가 있는 환자에게는 이 약의 투여를 피해야 한다.), 폐경색, 중증의 폐기능장애 환자 및 기타 저산소혈증을 수반하기 쉬운 상태, 과도한 알코올 섭취자, 탈수증, 설사, 구토 등의 위장장애 환자
9) 임부, 임신하고 있을 가능성이 있는 여성, 수유부

3. 다음 환자에게는 신중히 투여할 것 (유산산증과 저혈당의 가능성이 있다.).
1) 불규칙한 식사, 식사 섭취량 부족
2) 격렬한 근육운동
3) 상호작용이 있는 약물을 투여하는 환자

1.3 이상 반응

1) 유산산증 : 유산산증은 드물기는 하나 심각하며, 치료기간 중 이 약이 축적되어 나타나는 대사성 합병증이다. 발생한 경우에 있어서는 50%가 치명적이다. 유산산증은 조직의 유의한 관류저하와 저산소증이 있을 때, 당뇨병을 포함하는 병리생리학적 상태와 결부되어 나타날 수 있다. 유산산증은 혈중 젖산 농도 증가(5mmol/L 초과), 혈중 pH저하, 음이온 간의 차이 가 늘어나는 전해질 불균형과 젖산/피루브산염의 비가 증가하는 것을 특징으로 한다. 이 약이 유산산증의 원인이라고 여겨지는 경우, 이 약의 혈중농도는 일반적으로 5㎍/mL을 초과하는 것으로 나타난다. 이 약을 투여 받은 환자에 있어서 유산산증의 발현에 대한 보고는 매우 적다(연간 환자 1,000명당 약 0.03건, 치명적인 경우는 연간 환자 1,000명당 약 0.015건). 보고된 경우는 주로 많은 내. 외과적인 문제가 병합된 경우와 많은 약물의 복합투여 등의 상태에서, 내인적 신장질환과 신장 관류저하를 포함하는 신기능이 유의하게 저하된 당뇨병 환자에서 일차적으로 발생된 것이다. 유산산증의 위험은 신기능 저하의 정도와 환자의 나이에 따라 증가된다. 따라서 유산산증의 위험은 이 약을 투여하는 환자의 신기능을 정기적으로 모니터링하고, 최저 유효량을 투여함으로써 유의적으로 저하될 수 있다. 덧붙여 이 약은 저산소증, 탈수, 패혈증과 관계된 증상이 나타날 경우 즉각 투여를 중지해야 한다. 간기능이 손상된 경우 젖산 배설능력이 유의적으로 저하될 수 있으므로, 일반적으로 간질환의 임상적 또는 실험실적 소견이 있는 환자의 경우 이 약을 투여하지 않는다. 알코올은 이 약이 젖산대사에 영향을 미치게 할 가

능성이 있으므로, 이 약을 투여 받는 동안 급. 만성적인 알코올의 과량섭취는 피해야 한다. 또한, 혈관 내 방사선 조영제 검사나 수술 전에는 이 약의 투여를 일시적으로 중지해야 한다. 유산산증의 시작은 때때로 구별이 어렵고 권태, 근육통, 호흡곤란, 심해지는 졸음과 복부 통증과 같은 비특이적 증상들을 수반한다. 산증이 더 심해질 경우 체온저하, 저혈압과 저항성 서맥부정맥이 나타날 수 있다. 환자와 의사는 이러한 증상들의 중요성에 대해 알고 있어야 하며, 환자는 만약 이러한 증상이 나타나는 경우 의사에게 즉시 알려야 한다. 혈청 전해질, 케톤, 혈당, 혈중 pH, 젖산농도와 이 약의 혈중 농도도 유산산증을 확인하는데 유용할 수 있다. 또한, 유산산증의 의심이 큰 경우에는 젖산의 측정결과를 기대할 것 없이 필요한 처치를 한다. 특히, 투여 개시 초기 투여량을 증가한 경우에는 유산산증이 발생하기 쉬우므로 주의한다. 치료 초기에 환자가 이 약의 특정 용량에서 안정화되면 흔히 위장관 증상이 나타날 수 있으나, 후기에 나타나는 위장관 증상은 유산산증이나 다른 심각한 질병 때문일 수 있다. 이 약을 투여 받은 환자의 공복 시 정맥 혈장의 젖산 농도가 정상상한치 이상이지만 5mmol/L이하인 경우, 유산산증이 임박했다는 것을 나타내지 않으며, 잘 조절되지 않은 당뇨병이나 비만, 지나친 육체활동 또는 검체를 다루는데 있어 기술적인 문제와 같은 다른 기전에 기인할 수 있다. 유산산증은 케톤산증(케톤뇨증과 케톤혈증)의 증거가 없는 대사성 산증이 나타나는 당뇨환자에서 의심해야 한다. 유산산증은 입원치료를 요하는 내과적 응급상황이다. 이 약을 투여한 환자에서 유산산증이 일어난 경우, 약물을 즉시 투여 중지하고 즉각 일반적인 보조요법을 실시해야 한다. 이 약은 투석이 가능하기 때문에(양호한 혈역학적 조건에서 170mL/분에 이르는 청소율), 산증을 정상화하고 축적된 약물을 제거하기 위해 즉각적인 혈액 투석이 추천된다. 이러한 처치는 때때로 즉각적인 증상 호전과 회복을 가져온다.

2) 소화기계 : 위장관계 증상(설사, 구역, 구토, 복부팽만, 식욕부진, 소화불량, 변비, 복통)은 이 약 투여 시 나타나는 가장 일반적인 반응이며, 특히 치료의 초기에는 위약 투여군에 비해 이 약 단독 투여 군에서 약 30% 정도 빈번하게 발생한다. 이러한 증상은 일반적으로 일시적이며, 치료를 계속하는 동안 자연적으로 사라진다. 때때로, 일시적인 용량감소가 유용할 수 있다. 임상 시험에서는 약 4%의 환자가 위장관계 반응으로 이 약을 중지하였다. 치료초기 동안 위장관계 증상은 용량과 관련하여 발생되기 때문에 용량을 점차적으로 증가시키거나, 이 약을 식사와 함께 투여함으로써 감소시킬 수 있다. 심각한 설사 그리고/또는 구토는 탈수와 신전 질소혈증을 일으킬 수 있으므로, 이런 조건하에서는 일시적으로 중지하여야 한다. 이 약에 안정화가 이루어진 환자의 경우, 비특이적인 위장관계 증상은 치료에 기인한 것이 아니라 병발한 질병이나 유산산증의 가능성이 있다.

3) 감각기계 : 치료초기 동안, 약 3%의 환자가 불쾌하거나 금속성 미각을 호소하나, 보통 점차 소실된다.

4) 피부 : 발진 등이 나타날 수 있으므로 이러한 경우 투여를 중단한다.

5) 혈액계 : 드물게 빈혈, 백혈구 감소, 혈소판 감소가 나타날 수 있다. 29주 동안의 대조임상시험에서 이 약 단독요법의 약 9%환자와 이 약과 설폰요소 병용 요법의 약 6%의 환자에서 무증상으로 혈청 비타민 B12치가 정상이하로 감소하였으나 혈청 엽산 농도는 유의적으로 감소하지 않았다. 하지만 신경병증의 빈도는 증가되지 않고, 5건의 거대적아구성 빈혈이 이 약 투여와 관련하여 보고되었다(미국의 임상시험동안은 없었음). 따라서 혈청 B12값을 적절하게 모니터링하거나, 정기적으로 비경구적인 B12 보급을 고려해야 한다.

6) 간장 : 때때로 간기능 이상이 나타날 수 있다.

7) 저혈당 : 드물게 중증의 지연성 저혈당을 일으킬 수 있다.

8) 소아에서의 이상반응 : 소규모 소아 코호트인 10~16세 연령의 환자에 1년동안 메트포르민을 투여한 임상시험 뿐만 아니라 발표된 그리고 시판 후 조사결과에서 보고된 이상반응은 그 특성 및 심각도 측면에서 성인에서 보고된 이상반응과 유사했다.
